# Diffusion models for virtual populations and pharmacometric simulations

**DOI:** 10.1007/s10928-026-10054-7

**Published:** 2026-07-29

**Authors:** Prathamesh Kishor Gadgil, Shamith Manjunath Poojari, Murali Ramanathan

**Affiliations:** Artificial Intelligence & Clinical Pharmacology Laboratory, 355 Pharmacy, Department of Pharmaceutical Sciences, University at Buffalo, The State University of New York, Buffalo, NY 14214-8033 USA

**Keywords:** Diffusion models, Physiological determinants of drug dosing, Generative AI, Artificial intelligence, Pharmacometrics, Tabular VAE, Precision medicine

## Abstract

To evaluate diffusion model-based artificial intelligence approaches for generating virtual populations with physiological determinants of drug dosing (PDODD) and pharmacokinetic (PK) profiles. A denoising diffusion probabilistic model (DDPM) was applied to a 31-variable dataset of PDODD covariates (18 continuous, 13 binary) from the National Health and Nutrition Examination Survey and compared to a tabular variational autoencoder (TVAE). For nivolumab PK data (12,000 patients, 13 time points, 5 covariates), sequence-based diffusion model (SDM) and a time-aware diffusion model (TDM) with temporal self-attention were evaluated. The predictive performance of the TDM was evaluated by imputing masked time points. All models were trained and tested on 80%:20% partitions of the data using univariate, bivariate, and multivariate distributional similarity metrics. The diffusion model satisfactorily approximated the univariate distributions of continuous PDODD biomarkers (mean Kolmogorov-Smirnov D-statistic, KSD = 0.014), disease status frequencies (mean absolute error, MAE = 0.31%), and preserved bivariate correlations (MAE = 0.033). DDPM outperformed TVAE for categorical variables (0.31% vs. 1.07% MAE) and correlation (0.033 vs. 0.091 MAE). For nivolumab PK, SDM has KSD of 0.047 and a relative error of 1.36%. TDM accurately imputed missing PK timepoints (KSD = 0.014), reconstructing masked Day 1, Peak concentration (Cmax) Dose-9, and Terminal phase concentrations with MAE of 0.43%, 0.25%, and 0.76%, and correlations ≥ 0.999. Diffusion models demonstrated strong performance in generating cross-sectional PK covariate data and longitudinal PK profiles, capturing complex distributional and temporal dependencies. Diffusion-based approaches provide a flexible and robust framework for virtual simulations in pharmacometrics.

**Supplementary Information:**

The online version contains supplementary material available at https://doi.org/10.1007/s10928-026-10054-7.

## Introduction

Successful drug development relies on understanding how patient characteristics, disease biology, and pharmacokinetic-pharmacodynamic (PK-PD) relationships interact to influence drug response. Modeling approaches such as population PK, physiologically based PK (PBPK), and quantitative systems pharmacology (QSP) models can be limited in their ability to integrate diverse, high-dimensional datasets containing multiple physiological biomarkers and disease variables. Artificial intelligence (AI) methods are increasingly transforming clinical pharmacology by enabling the integration of large, heterogeneous datasets and supporting complex decision-making in drug development [1].

Recent advances in AI, including generative adversarial networks (GANs) [2]and variational autoencoders (VAEs) [3], which use deep neural networks, have been shown to be useful for virtual populations [1]. GANs employ adversarial training via classification, whereas VAEs perform nonlinear dimensionality reduction. GANs and VAEs are new generative AI modeling paradigms that can learn the high-dimensional joint distribution describing relationships among biomarkers and key patient covariates from real-world data [4–6]. Random variate vectors sampled from the learned joint distribution can generate virtual patient populations with physiological and disease biomarkers, enabling more realistic and informative clinical trial simulations. High-quality synthetic data is particularly useful in clinical settings, such as rare diseases and underrepresented groups, where trial recruitment is challenging.

Our group has investigated GAN architectures, specifically conditional tabular GANs (CTGANs) for generating virtual population datasets of physiological determinants of drug dosing (PDODD) [5]and for racial differences in metabolic biomarkers using data from the National Health and Nutrition Examination Surveys (NHANES) [4]. Jiang et al. compared PK/PD datasets generated by CTGANs to those generated by Time-series GANs and probabilistic autoregressive models [7]. Rohleff et al. introduced several innovations in the VAE framework relevant to pharmacometrics, including the use of a long short-term memory layer in the encoder, modification of the evidence lower bound objective to accommodate longitudinal mixed data with both within- and between-subject variability, and application of a corrected Bayesian information criterion for covariate selection in theophylline data [8].

The “traditional” statistical approaches for obtaining virtual populations, such as resampling, Bayesian modeling, and copula-based methods, have limitations when applied to high-dimensional data sets [5]. Resampling methods simply provide random subsets of the input data; Bayesian models require prior distributions and hyperpriors, and copula methods require the marginal distributions of each variable as well as a copula function to define the correlation structure among variables [5].

There are key advantages of generative AI methods over existing pharmacometric approaches, such as nonlinear mixed-effects modeling (NLME), PBPK models, and emerging approaches such as QSP models and Bayesian methods. NLME models, which are widely used in drug development, rely on compartmental approaches for structural modeling, additive or multiplicative terms for covariate effects on parameters, and a linear error term on outputs. NLME models require users to specify equations and parameters for all structural, covariate, and error relationships, limiting their utility for high-dimensional datasets with complex dependencies. PBPK and QSP models, which incorporate numerous prespecified parameters and complex interdependencies that appear biologically plausible, pose substantial identifiability challenges and often lack generalizability across drugs and disease states. Bayesian methods require selecting prior distributions for all structural, covariate, and error parameters and entail high computational costs.

Unlike these statistical and pharmacometrics methods, generative AI approaches can learn and approximate the high-dimensional joint distribution of PDODD from data. Users do not have to provide a structural, covariate, or error model as input. Generative AI methods require more data but enable a more comprehensive statistical characterization of trends, pairwise correlations, multivariate relationships, and inter-individual variability among its constituent variables [5]. Yang et al. review the pros and cons of generative AI methods vis-à-vis legacy in silico approaches [1].

Diffusion models are a powerful class of generative models [9] that have been used for image generation, molecular modeling for discovery, and temporal data modeling for time-series imputation, forecasting, and signal processing [10]. Yang et al. review the foundations of diffusion models, similarities and differences with GANs, VAEs, and time-series models, as well as their applications [10]. Like GANs and VAEs, diffusion models were developed for image synthesis but are being adapted to other domains requiring complex generative capabilities. Diffusion models work by learning to iteratively reverse a stochastic noise process applied to data, allowing for the reconstruction or generation of realistic samples from an underlying probability distribution. Diffusion models can capture complex, nonlinear relationships in high-dimensional data while remaining stable during training, and their iterative generation process enables explicit conditioning on constraints or prior knowledge, making them especially suited for pharmacometrics. However, diffusion models have not been extensively investigated in the context of clinical pharmacology and pharmacometrics.

The objectives of this research are to: (i) evaluate diffusion models for generating tabular datasets representing physiological determinants of drug dosing and pharmacokinetic (PK) profiles in disease states within PDODD; (ii) compare their performance with VAEs; (iii) evaluate diffusion modeling for generating PK concentration-time profiles and covariates; and iv) assess sequence-based (SDM) and time-aware modeling (TDM) strategies; SDM provides the sequence of concentrations as input, whereas TDM includes time-concentration pairs as input. The TDM strategy could support training with data sets containing different sampling patterns and missing time points.

## Methods

### Generative diffusion modeling of PK covariates

#### Diffusion architecture

**Fig. 1:**
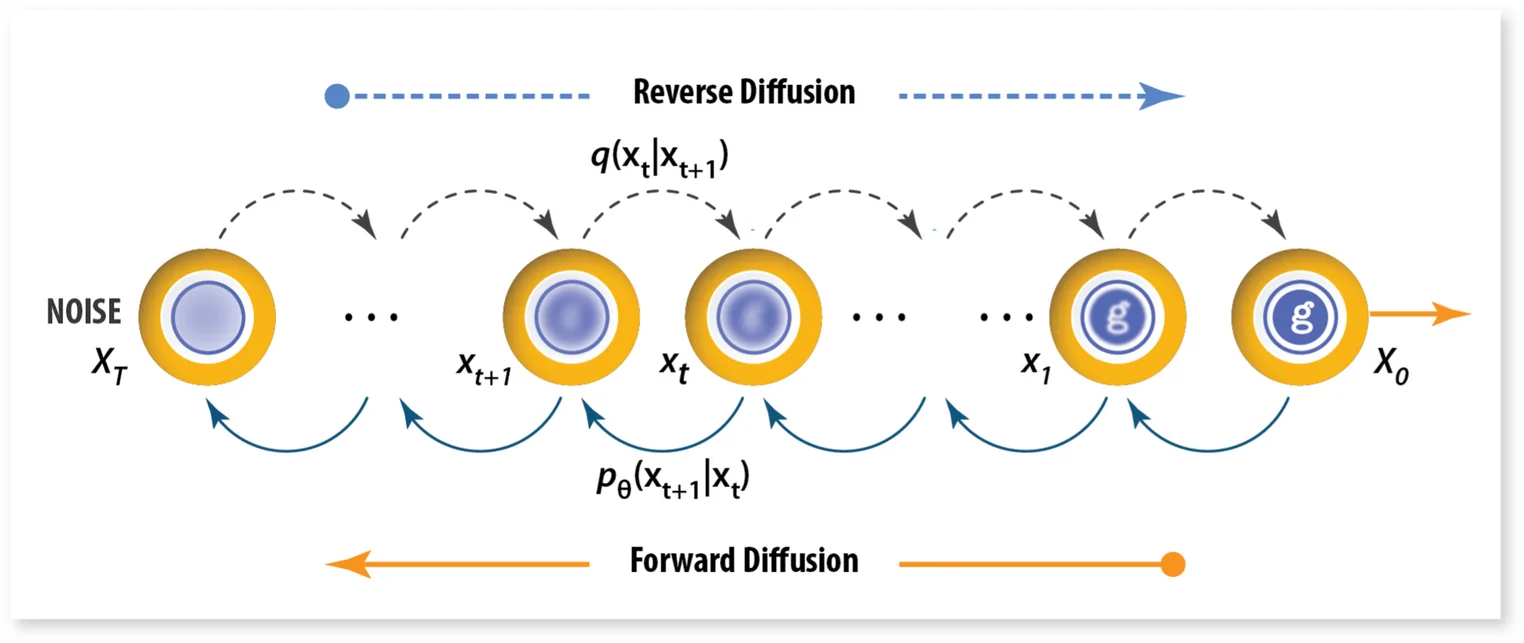
Schematic representation of a diffusion model. Diffusion models consist of forward and reverse diffusion processes. In forward diffusion, Gaussian noise is sequentially added to a data distribution, gradually corrupting it, and at infinite time, the data becomes pure noise. In reverse diffusion, a neural network is trained to remove this noise and recover a new data distribution

Figure 1 is a schematic of the diffusion model architecture. Diffusion models are a class of generative AI methods that learn to produce new data by reversing a gradual noise-corruption process.

The approach consists of two coupled processes: a forward diffusion process and a reverse denoising process. In the forward process (Fig. 1, bottom arrows), Gaussian noise is sequentially added to the training data x₀ over T timesteps, producing progressively noisier versions $x_1,\;x_2,...,\;x_T$ until the data distribution is transformed into pure noise. The reverse process (Fig. 1, top arrows) trains a neural network parameterized by $\:\theta\:$ to recover the data by learning the conditional transition $\:{p}_{\theta\:}\left({x}_{t+1}\right|{x}_{t})$ at each step. Once trained, new synthetic data can be generated by starting from random noise $\:{x}_{T}$ and iteratively denoising through the learned reverse transitions to produce a sample from the data distribution.

The forward process followed a denoising diffusion probabilistic model (DDPM) framework with a linear noise schedule over 1000 timesteps, where the variance parameter β increased linearly from 0.0001 to 0.02. At each timestep * t*, a noised version of the input $\:{x}_{t}$ is computed from the original data $\:{x}_{0}$ and Gaussian noise ε using a closed-form expression governed by the cumulative product of the noise schedule. This formulation permits direct sampling of $\:{x}_{t}$ at any arbitrary timestep without requiring sequential computation of all preceding steps.

The denoising network was a multi-layer perceptron (MLP) with residual connections that accepted the noised data vector and a representation of the current timestep as inputs and produced a prediction of the noise component to be removed. Timestep information was encoded using sinusoidal position embeddings, an approach adapted from transformer architectures.

The network comprised four hidden layers with dimensions of 256, 512, 512, and 256 units, respectively. Each residual block incorporated layer normalization, sigmoid linear unit (SiLU) activation functions, and dropout regularization at a rate of 0.1.

The training objective was to minimize the mean squared error (MSE) between the noise predicted by the network and the actual Gaussian noise added during forward diffusion. Continuous variables were recovered through inverse ordered quantile (inverse ORQ) normalization, while categorical variables were obtained by applying a threshold of 0.5 to the generated values.

To address class imbalance present in several disease status variables, such as dialysis at (0.1% positive) and hepatitis B (0.6% positive), class-specific weights derived from inverse frequency ratios were incorporated into the loss computation for the categorical variable dimensions.

The model was trained using the Adam optimizer with a learning rate of 0.0001 and weight decay of 0.0001. A cosine annealing learning rate schedule with a 10-epoch warmup period was employed. An exponential moving average (EMA) of the model weights was maintained throughout training with a decay factor of 0.9999 and was used during sample generation to improve the stability and quality of synthetic outputs. Training proceeded for up to 500 epochs with a batch size of 256, and early stopping with a patience of 50 epochs was applied to prevent overfitting.

#### Stability analysis

The stability of the trained diffusion model was evaluated by comparing the univariate density functions of the continuous and disease-status variables across five generated datasets.

#### Tabular VAE

For comparison, the tabular VAE (TVAE) investigated in earlier PDODD work by Titar and Ramanathan [6] was evaluated for the NHANES dataset. The TVAE was implemented using the Synthetic Data Vault (SDV) Python library (https://docs.sdv.dev/sdv/). To enable fair comparison, TVAE was trained on the same ORQ-normalized data and train-test partition used for the diffusion model.

Hyperparameters followed those reported in [4]: encoder and decoder dimensions of (512, 512), embedding dimension of 128, batch size of 2000, L2 regularization scale of 10⁻⁷, loss factor of 4, and Kullback-Leibler annealing from 0 to 1 over 100 epochs. Training was conducted for 1000 epochs using the Adam optimizer with a learning rate of 0.001 and the evidence lower bound (ELBO) loss function.

#### Data source

Data were obtained from five consecutive cycles (2009–2010, 2011–2012, 2013–2014, 2015–2016, and 2017–2018) of the National Health and Nutrition Examination Survey (NHANES), a nationally representative cross-sectional survey of the United States population with laboratory measurements, physical examination, and questionnaire data [11]. NHANES data were accessed and compiled using the*nhanesA*R package [12].

#### Selected variables

The modeled biomarker panel comprised 31 variables: 18 continuous biomarkers and 13 binary disease-status indicators related to drug disposition. A subset of biomarkers was obtained directly from the NHANES dataset, and the remaining were calculated from combinations of variables using the equations summarized in the Supplementary File.

The 18 continuous biomarkers included age at screening (RIDAGEYR, years), and anthropometric measures: body weight (BMXWT, kg), normalized waist circumference (WAISTMF), body surface area (BSA, m²), along with blood composition markers: plasma volume (PLASMAVOL, liters), which are determinants of drug volume of distribution. Serum albumin (LBXSAL, g/dL) was included because it binds of acidic and neutral compounds and can affect drug free fraction. Hepatic and renal biomarkers were included because these are the liver and kidney are the major organs responsible for drug metabolism and clearance. Hepatic function was represented by the hepatic R-value (RVALUE), drug-induced liver injury index (DILI), and hepatic steatosis index (HSI). Renal markers included urine flow rate (URDFLOW, mL/min), urine creatinine (URXUCR, mg/dL), urine albumin-creatinine ratio (URDACT, mg/g), and estimated glomerular filtration rate (EGFR, mL/(min 1.73 m²)). Red blood cell count (LBXRBCSI, 10⁶ cells/µL), platelet count (LBXPLTSI, 10³ cells/µL), lymphocyte count (LBDLYMNO, 10³ cells/µL), segmented neutrophil count (LBDNENO, 10³ cells/µL), and the systemic inflammation index (SII) derived from complete blood count with differential and platelets were included because drug-induced cytopenias are common hematological adverse effects.

The 13 binary disease status variables were related to renal impairment (kidney disease, dialysis), hepatic conditions (active liver disease, past liver disease, active hepatitis B, active hepatitis C), metabolic status (diabetes, prediabetes or diabetes, insulin use), and cardiac disease (heart attack, congestive heart failure, coronary heart disease, angina pectoris).

#### Data pre-processing

Subjects aged 20 years and older were included in the analysis. Individuals aged ≥ 80 years were recorded as 80 years old. Listwise deletion was used to obtain complete cases.

The 13 disease status variables were encoded as numeric binary variables (1 = Positive, 0 = Negative). Continuous biomarkers were logarithmically (base-10) transformed to reduce skew. Serum albumin and estimated glomerular filtration rate were not log-transformed as their distributions were sufficiently symmetric; a small positive constant (10^–4^) was added to address zero values present in the DILI. The resulting predictor variable set was then ORQ normalized [13]. ORQ scaling yields normal distributions with zero mean and unit standard deviation, and the transformation can be reversed to recover the original variable values.

The dataset was 80:20 divided into training (*n* = 13,984) and test (*n* = 3,496) subsets using stratified random sampling by diabetes status to preserve the class distribution across partitions.

### Generative diffusion modeling of PK trajectories

Two approaches, sequence-based diffusion (SDM) and time-aware diffusion (TDM) modeling, were evaluated for generative modeling of PK trajectories. SDM was provided with nivolumab data vectors containing concentration sequences and covariates as inputs; time point information was not included. The TDM was provided with time-concentration pairs and covariates as input.

#### SDM architecture

The same DDPM architecture described in the “Generative Modeling of PK Covariates” section was applied to the nivolumab dataset with 18 input features. To address the imbalance between the 16 continuous and two binary dimensions, dimension-specific loss weights were applied, with binary dimensions receiving a weight of 15 relative to continuous dimensions, normalized to unit mean. A cosine annealing learning rate schedule decayed from 0.001 to 0.00002 over 800 training epochs.

#### TDM architecture

The TDM architecture was based on an adaptation of the diffusion approach described by Zheng et al. for time-series imputation [14]. The TDM employed temporal self-attention across the 13 PK timepoints, allowing each timepoint’s representation to attend to all others during denoising.

The architecture consisted of four transformer-style blocks, each comprising multi-head self-attention (4 heads), layer normalization, and a feed-forward network with GELU activation and a hidden dimension of 128. Temporal position encoding was implemented by applying ordered quantile normalization to the log-transformed sampling times, producing evenly spaced position values that encoded the irregular time spacing of the clinical sampling schedule (e.g., 1-hour post-dose vs. Day 322).

Patient covariates were incorporated via a dual-conditioning mechanism. A global covariate embedding, produced by a three-layer MLP, was fused with sinusoidal diffusion timestep embeddings and added to all timepoint representations. Additionally, a per-timepoint covariate projection generated distinct conditioning vectors for each of the 13 timepoints, enabling the model to learn timepoint-specific covariate effects, e.g., the influence of body weight on peak concentration (Cmax) vs. terminal-phase concentration.

The input to the denoiser at each time point consisted of two channels: the noised concentration value and a binary mask indicating whether the position was observed (mask = 1) or to be generated (mask = 0). During training, a mixed masking strategy was employed: 30% of batches used full masking (all 13 timepoints masked), training the model for de novo generation, while the remaining 70% used random partial masking (20–80% of timepoints masked), training the model for imputation from observed data. The loss was computed as the mean squared error between predicted and actual noise, restricted to masked positions in partially masked batches and applied to all positions in fully masked batches.

The diffusion process utilized the same linear noise schedule as SDM (β from 0.0001 to 0.02). The model was trained for 2000 epochs using the Adam optimizer with cosine annealing (learning rate from 0.001 to 0.00002). At inference, synthetic trajectories were generated by starting from Gaussian noise at all 13 time points and performing the full 1000-step reverse diffusion conditioned on sampled covariates.

The predictive performance of the TDM was evaluated with an imputation test. Three timepoints spanning different PK phases were masked in the test set: Day 1, Cmax D-9 (112.0416 days), and Terminal (322 days). The model predicted the masked concentrations from the 10 remaining observed time points and patient covariates using the same reverse diffusion process with observed values re-imposed at each denoising step.

#### Dataset from nivolumab population PK simulations

A simulated nivolumab pharmacokinetic (PK) dataset was generated from a two-compartment population PK model with time-varying clearance [15] using*nlmixr2*[16, 17]. Nivolumab was administered via intravenous infusion at 3 mg/kg every two weeks (Q2W) for 12 consecutive doses. The R code for the model was obtained from PK_2cmt_tdcl_des from the*nlmixr2lib*package [18]; the model parameters are in (See Supplementary Table S1).

The model was extended to include covariate effects, as described in Bajaj et al. [19]. The simulated population comprised 12,000 virtual patients with five covariates: body weight (WT, kg), serum albumin (ALB, g/dL), tumor volume (TUM), sex (SEX), and ECOG performance status (ECOG).

From the full concentration-time profiles, 13 time points (See Supplementary Table S2) reflecting a typical clinical trial sampling schedule were selected: Cmax D-1 (1 h, at end of infusion), Day 1, Day 3, and Cmax (at end of infusion), mid-cycle (7 days after start of infusion), and trough concentrations (14 days after start of infusion) time points at Cycles 6 (Day 70), 9 (Day 112), and 12 (Day 154), along with a terminal-phase sample (Day 322).

#### Data preprocessing

The covariates and nivolumab concentrations for each subject were reshaped into a vector with 18 features (13 concentrations, 3 continuous covariates, and 2 binary covariates) for SDM with concentrations and continuous covariates logarithmically (base-10) transformed and ordered-quantile normalized based on the training partition.

For TDM, we included time values with corresponding concentrations and covariates (13 time-concentration pairs = 26 variables; 3 continuous covariates; and 2 binary covariates). Time values and continuous covariates were logarithmically (base-10) transformed and ordered-quantile normalized. Nivolumab concentrations were logarithmically (base-10) transformed, and globally ordered-quantile normalized, with a single quantile transformation fit across all timepoints pooled. Binary covariates were passed through without transformation.

The dataset was divided into training (*n* = 10,000) and test (*n* = 2,000) subsets using stratified random sampling by sex.

### Data analysis

Model implementation, training, and synthetic data generation were conducted in Python 3.9 using PyTorch 2.0, with scikit-learn for ordered quantile normalization (QuantileTransformer) and NumPy/Pandas for data manipulation. The TVAE model was implemented using the Synthetic Data Vault (SDV) Python library. All model training was performed on a single NVIDIA A100 GPU on the University at Buffalo Center for Computational Research (CCR) high-performance computing cluster.

Evaluation of the generated data, statistical comparisons, and visualizations were performed using the R statistical programming environment [20, 21].

#### Generated data evaluation

The joint distributions of the generated data from the diffusion and TVAE models were compared to the test data distribution using a variety of metrics.

#### Univariate distributions

For disease status variables, bar charts of positive-class frequencies were used.

Marginal distributions of categorical and binary variables in the real (R) and synthetic (S) datasets were compared using the total variation complement (TVC) [22]:

$$\:TVC=1-\frac{1}{2}\left(\sum\:_{{\upomega\:}\in\:{\Omega\:}}\left|R\left({\upomega\:}\right)-S\left({\upomega\:}\right)\right|\right)$$

where ω denotes the elements in the event space Ω.

Probability density histograms were used to visually compare the marginal distributions of each continuous biomarker in the test and diffusion-generated data. Distributional agreement was assessed using the Kolmogorov-Smirnov complement (*KSC*) [23], expressed in terms of the Kolmogorov-Smirnov statistic (*KSD*):

$$\:KSC=1-KSD\left(R,S\right)$$

Both TVC and KSC are bounded between 0 and 1, where values approaching 1 indicate close agreement and values near 0 indicate substantial dissimilarity between the two distributions.

#### Bivariate distributions

Correlation heat plots were used to visualize the bivariate Pearson correlation coefficient patterns between continuous variables in the test vs. diffusion model-generated data. A difference heatmap was also computed to identify variable pairs. Bivariate scatter plots with marginal densities and pairwise correlation coefficients for a subset of continuous variables were obtained using the *GGally* R package.

#### Multivariate distributions: dissimilarity measures for multivariate distributions

The nonparametric maximum mean discrepancy (MMD) test was computed using the *kernlab*R package [24] with automatic bandwidth selection for the Gaussian radial basis function kernel.

Two-dimensional projections for visual assessment of the high-dimensional joint distribution were obtained with the *t*-distributed stochastic neighbor embedding (t-SNE), uniform manifold approximation and projection (UMAP), and principal component analysis (PCA) algorithms computed with the *Rtsne*, *umap*, and *prcomp*R packages, respectively [25, 26].

### Comparisons of exposure measures and covariate-exposure relationships

We assessed exposure measures and covariate-exposure relations for the test and generated datasets from the TDM model. Trough concentrations at Dose 6, Dose 9, and Dose 12 were assessed as the exposure measures. The covariate relationships of sex, ECOG performance status, tumor volume (TUM), and body weight (WT) were evaluated using box plots for discrete variables and loess-fitted scatter plots for continuous variables (WT and TUM).

## Results

### Generative diffusion modeling of PK covariates

#### Characteristics of study subjects

The initial data set consisted of *n* = 23,825 subjects, and *n* = 17,480 (73.4%) complete observations were retained following listwise deletion. The frequency of missing data among the continuous biomarkers ranged from 5.5% for body weight to 13.3% for urine flow rate. The frequency of missing data for the categorical disease status variables ranged from 0.04% for insulin use to 5.6% for active liver disease.

Table 1 presents summary statistics for demographic characteristics, continuous biomarker levels, and disease status frequencies. The frequency of females in the retained sample was 51.9%, and the mean age (SD) was 49.2 (17.3) years.

**Table 1:** Summary of the demographic characteristics and drug disposition biomarkers

Disease Status	Negative	Positive
Dialysis	17,457 (99.9)	23 (0.1)
Kidney Disease	16,925 (96.8)	555 (3.2)
Active Liver Disease	17,053 (97.6)	427 (2.4)
Past Liver Disease	17,200 (98.4)	280 (1.6)
Hepatitis C	17,291 (98.9)	189 (1.1)
Hepatitis B	17,367 (99.4)	113 (0.6)
Diabetes	15,194 (86.9)	2286 (13.1)
Prediabetes	13,455 (77.0)	4025 (23.0)
Insulin Use	16,845 (96.4)	635 (3.6)
Heart Attack	16,819 (96.2)	661 (3.8)
Congestive Heart Failure	16,973 (97.1)	507 (2.9)
Coronary Heart Disease	16,821 (96.2)	659 (3.8)
Angina Pectoris	17,087 (97.8)	393 (2.2)
Abbreviation	Biomarker	Mean (SD)
RIDAGEYR	Age, years	49.2 (17.3)
BMXWT	Body weight, kg	81.7 (21.5)
WAISTMF	Normalized waist circumference	1.06 (0.19)
BSA	Body surface area, m^2^	1.89 (0.26)
PLASMAVOL	Plasma volume, L	4.28 (0.85)
LBXSAL	Albumin, g/dl	4.23 (0.35)
RVALUE	Hepatic R-value	1.61 (1.18)
DILI	Drug-induced liver injury index, x 1000	1.74 (5.51)
SII	Systemic inflammation index	511 (327)
HSI	Hepatic steatosis index	38.3 (8.06)
URDFLOW	Urine flow, ml/min	1.12 (1.43)
URXUCR	Urine creatinine, mg/dl	124 (81.8)
URDACT	Urine albumin to creatine ratio, mg/g	44.0 (341)
EGFR	Glomerular filtration rate, ml/(min 1.73 m^2^)	95.1 (22.2)
LBXRBCSI	Red blood cell count, 10^6^ cells/µl	4.67 (0.50)
LBXPLTSI	Platelet count, 10^3^ cells/µl	238 (61.4)
LBDLYMNO	Lymphocyte count, 10^3^ cells/µl	2.20 (2.99)
LBDNENO	Segmented neutrophil count, 10^3^ cells/µl	4.23 (1.72)

#### Diffusion model results

The diffusion model was trained for 202 epochs before early stopping was triggered, achieving a best validation loss of 0.2495. Training required approximately 3 min on a single NVIDIA A100 GPU.

#### Univariate continuous distributions

**Fig. 2:**
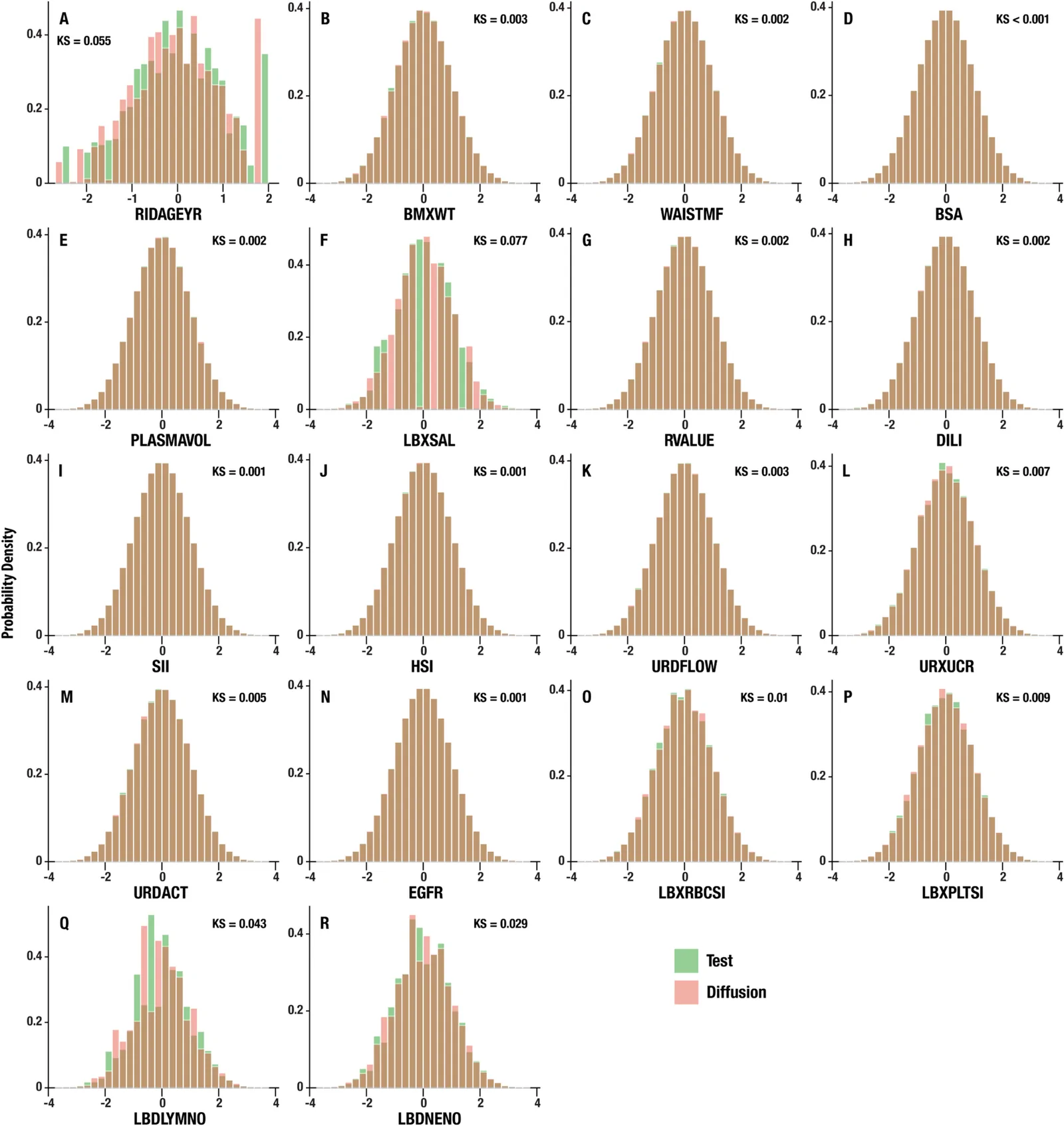
Probability density histograms of generated data from the diffusion model (salmon bars) compared to test data (green bars). The darker regions of the histogram bars correspond to the regions of overlap. The ordered quantile normalized values of log-transformed of the continuous biomarkers were plotted and are: age (RIDAGEYR, Figure 2**A**), body weight (BMXWT, Figure 2**B**), waist circumference normalized (WAISTMF, Figure 2**C**), body surface area (BSA, Figure 2**D**), plasma volume (PLASMAVOL, Figure 2**E**), serum albumin (LBXSAL, Figure 2**F**), hepatic R-value (RVALUE, Figure 2**G**), DILI value (DILI, Figure 2**H**), systemic inflammation index (SII, Figure 2**I**), hepatic steatosis index (HSI, Figure 2**J**), average urine flow (URDFLOW, Figure 2**K**), urine creatinine (URXUCR, Figure 2**L**), urine albumin to creatinine ratio (URDACT, Figure 2**M**), glomerular filtration rate (EGFR, Figure 2**N**), red cell number (LBXRBCSI, Figure 2**O**), platelet number (LBXPLTSI, Figure 2**P**), lymphocyte count (LBDLYMNO, Figure 2**Q**), and neutrophil count (LBDNENO, Figure 2**R**). The *x*-axes on all graphs are ordered quantile-normalized biomarker levels that are log-transformed

The univariate marginal distributions of the 18 continuous biomarkers generated by the diffusion model were compared with the test data. Figure 2 displays the probability density histograms of the diffusion-generated data (red bars) overlaid on the test data (green bars) for the ORQ of each biomarker. Extensive overlap between the two distributions was observed, indicating satisfactory approximation of the univariate marginal distributions.

The Kolmogorov-Smirnov D-statistic (KSD) values were low across all variables, with a mean of 0.014. Fourteen of the 18 biomarkers achieved *KSD* values < 0.01, including body surface area (0.0003), estimated glomerular filtration rate (0.0009), systemic inflammation index (0.0011), and hepatic steatosis index (0.0014). The remaining variables showed modestly higher but still satisfactory *KSD* values: neutrophil count (0.029), lymphocyte count (0.043), age (0.055), and serum albumin (0.077). The serum albumin distribution showed the largest departure from the test data, likely due to the apparently discrete nature of the measured values, recorded in 0.1 g/dL increments.

#### Univariate distributions of disease status variables

**Fig. 3:**
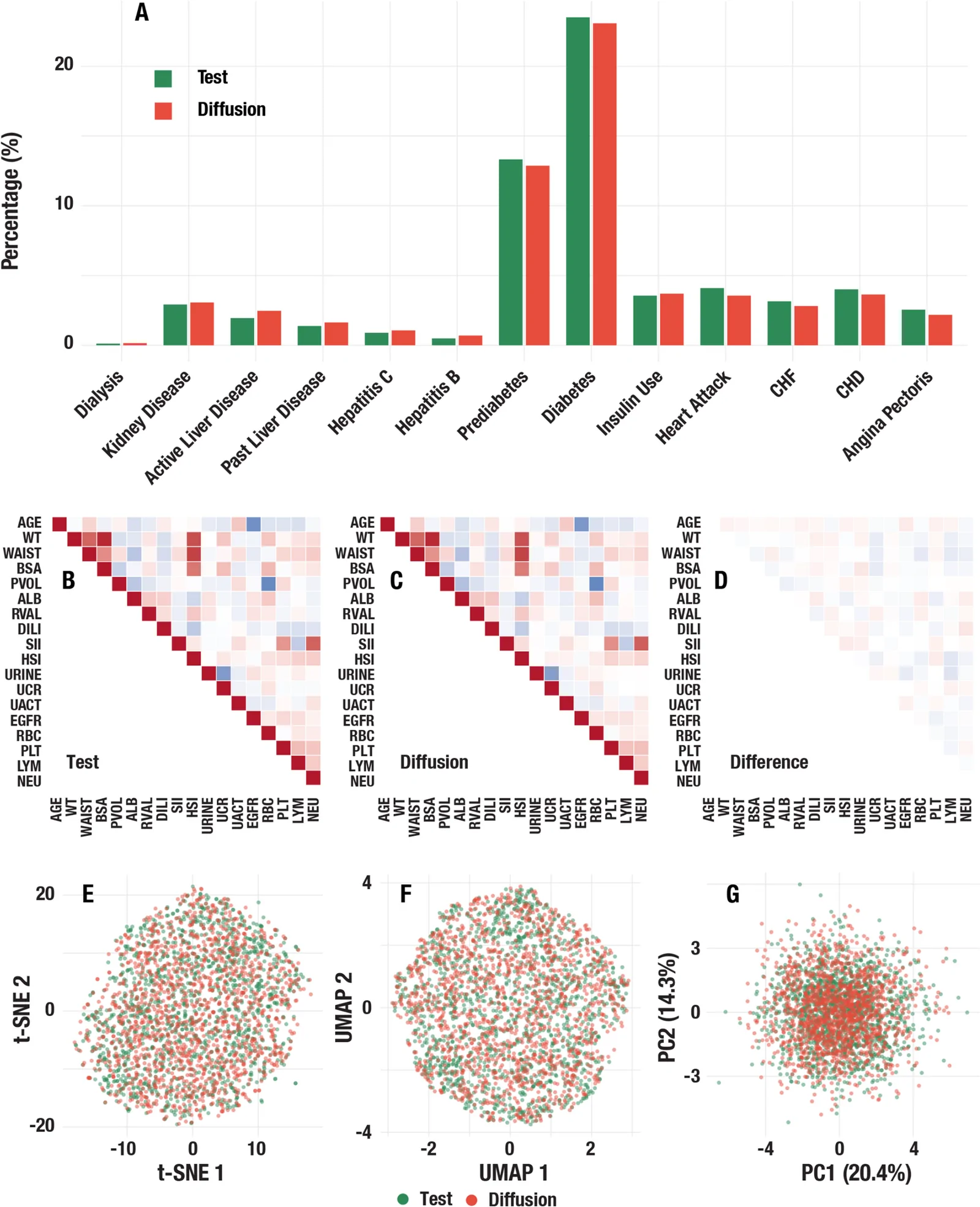
Figure 3**A** compares the percentages of the categorical variable shown on *x*-axis in the test (green bars) and diffusion-generated (salmon bars) data sets. The upper-triangular matrices of the correlograms for the test data, generated data, and the difference correlograms (Figure 3**C**) are shown in Figure 3**B**-**D**. The*t*-stochastic neighbor embedding (*t*-SNE, Figure 3**E**), uniform manifold approximation and projection (UMAP, Figure 3**F**), and principal component analysis (PCA, Figure 3**G**) are two-dimensional projections of the data. The test data results are shown in salmon circles, and the diffusion-generated results are in teal circles. The *x*-axis (t-SNE X and UMAP X) and *y*-axis (t-SNE Y and UMAP Y) correspond to the *t*-SNE and UMAP 2-dimensional projections of the input log-transformed, order-quantile normalized biomarker levels. PC 1 and PC 2 on the *x*-axis and *y*-axis of Figure 3**G** correspond to the first and second principal components, respectively; the proportion of the variance explained by each component is shown on the axes

The frequency distributions of the 13 binary disease status variables generated by the diffusion model are compared with the test data in Fig. 3 (top panel). The diffusion model closely reproduced the observed disease prevalences, with a mean absolute error of 0.31% across all categorical variables.

The low-frequency disease status variables were also satisfactorily approximated: dialysis (Test = 0.11%, Generated = 0.14%), hepatitis C (Test = 0.89%, Generated = 1.06%), and hepatitis B (Test = 0.49%, Generated = 0.69%). The largest discrepancy was observed for active liver disease (Test = 1.95%, Generated = 2.46%, Absolute error = 0.51%). The more prevalent conditions, such as diabetes (Test = 13.3%, Generated = 12.9%) and prediabetes (Test = 23.5%, Generated = 23.1%), were closely approximated.

#### Bivariate distributions

Correlation heatmaps were used to compare Pearson correlation coefficients among pairs of 18 continuous variables (Fig. 3, middle panel). The bivariate correlation patterns observed in the test data were satisfactorily estimated in the diffusion-generated data (Mean absolute correlation difference = 0.033). The difference heatmap (Fig. 3, middle panel) is consistent with this assessment.

The bivariate pairs plot for a subset of eight continuous variables (Supplementary Figure S1) additionally confirmed that the diffusion model preserved both the marginal distributions (diagonal panels) and the bivariate trends (lower panels). The pairwise correlation coefficients displayed in the upper panels showed close agreement between the test data (green) and diffusion-generated data (red), with the largest discrepancy observed for the URDACT–URDFLOW pair (Test = −0.093, Generated = −0.127).

#### Conditional distributions of biomarkers in disease states

Supplementary Figure S2 presents box plots of EGFR, urine albumin-creatinine ratio, and plasma volume across all 13 disease status categories for the test data (green boxes) and diffusion-generated data (red boxes). The diffusion model reproduced the expected disease-related shifts in biomarker levels. For most disease-biomarker combinations, the medians, interquartile ranges, and notch intervals of the generated data overlapped the test data distributions. The generated conditional distributions showed greater variability deviations in disease groups with small sample sizes (e.g., dialysis, with only 23 positive cases). Overall, the diffusion model captured the dependence structure between continuous biomarkers and binary disease indicators.

#### Diffusion-generated high-dimensional joint distributions

The t-SNE, UMAP, and PCA dimensionality reduction methods were utilized to obtain 2-dimensional projections of the high-dimensional joint distribution (Fig. 3, bottom panel). In all three visualizations, the diffusion-generated data (red points) overlapped extensively with the test data (green points), indicating that the high-dimensional joint distribution was satisfactorily approximated. The PCA projection showed that the first two principal components explained 20.4% and 14.3% of the variance, respectively, and the diffusion-generated data occupied the same region of the principal component space as the test data.

The maximum mean discrepancy (MMD) test yielded a value of 0.006, indicating close agreement between the multivariate distributions of the test and diffusion-generated data.

#### Stability analysis

The stability of the trained diffusion model was evaluated by comparing the univariate probability density histograms for five instances of generated data. Supplementary Figure S2 shows that the five histograms were concordant with each other and the test distribution.

#### Comparison of diffusion vs. TVAE models

The diffusion model was compared to TVAE on the same ORQ-normalized data and train-test partition.

Both models provided comparable univariate approximations for continuous biomarkers with mean KSD values of 0.065 for the diffusion model vs. 0.054 for the TVAE model. TVAE had lower KSD values for 10 of 18 continuous variables; the diffusion model had lower KSD for the remaining eight.

The diffusion model outperformed TVAE in reproducing the categorical disease status variable frequencies. The mean absolute error was 0.31% for the diffusion model vs. 1.07% for TVAE. The diffusion model achieved lower error for 12 of 13 categorical variables. The difference was most pronounced for lower frequency disease categories, e.g., kidney disease (Test data = 2.92%, Diffusion = 3.06%; TVAE = 0.94%) and active liver disease (Test data: 1.95%, Diffusion = 2.46%, TVAE = 0.14%).

The diffusion model was superior at preserving the pairwise correlation structure. The mean absolute correlation difference was 0.033 for diffusion vs. 0.091 for TVAE.

### SDM for generation of nivolumab PK trajectories

The SDM was trained on the 18-dimensional nivolumab dataset for 800 epochs, achieving a best training loss of 0.082 in approximately 7.7 min on a single GPU.

**Fig. 4:**
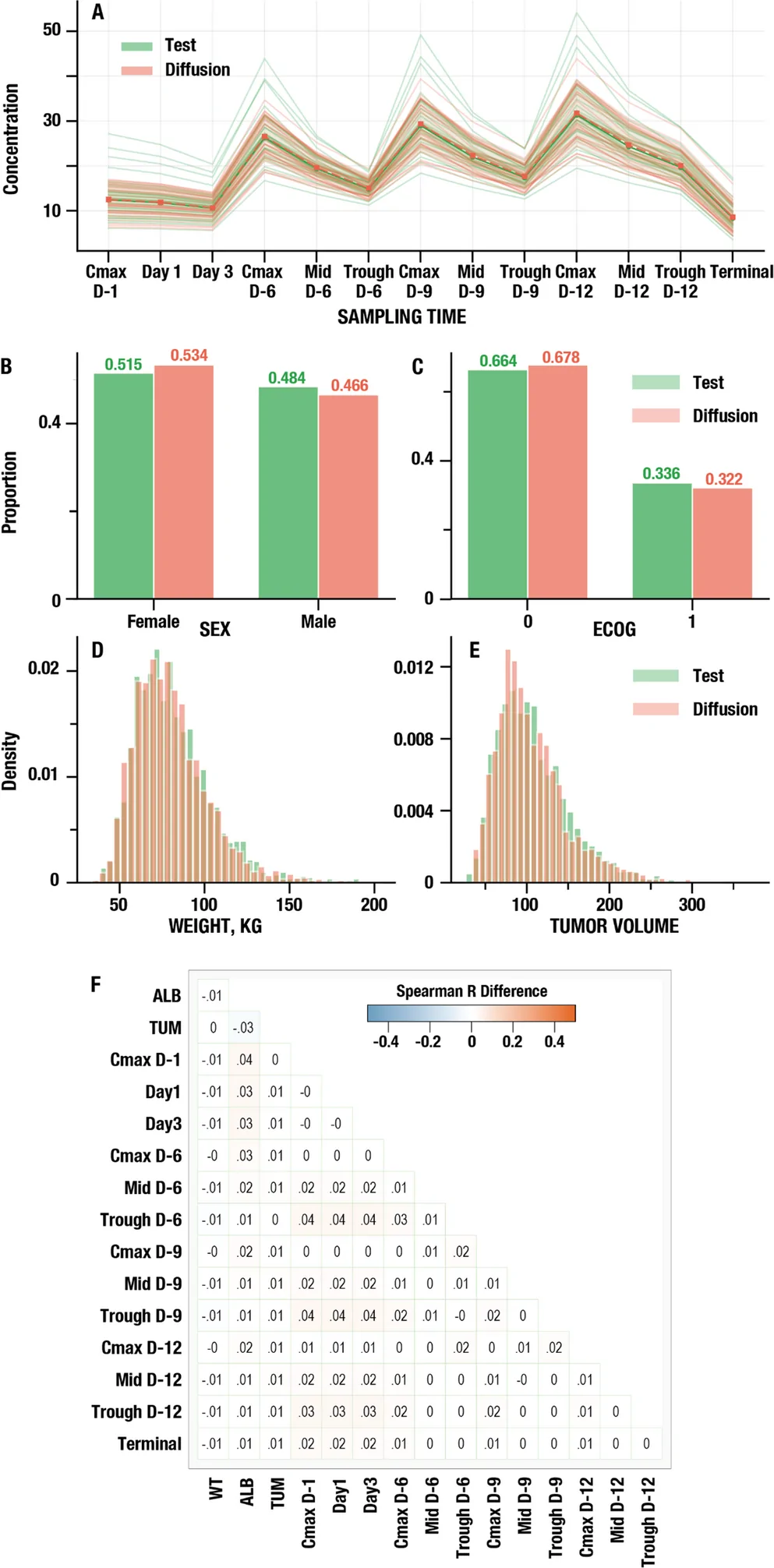
Figure**4**A shows the trajectory of nivolumab concentrations for 30 subjects each in the test (green lines) and sequence-based diffusion model (SDM) generated (salmon lines) data sets at the following time points: Cmax D-1 (Cycle 1 at end of infusion), Day 1, Day 3, Cmax D-6 (Cycle 6 at end of infusion), Mid Dose-6 (Mid D-6), Trough Dose-6 (Trough D-6), Cmax D-9 (Cycle 9 at end of infusion), Mid Dose-9 (Mid D-9), Trough Dose-9 (Trough D-9), Cmax D-12 (Cycle 12 at end of infusion), Mid Dose-12 (Mid D-12), Trough Dose-12 (Trough D-12), and Terminal phase concentrations. The dark lines correspond to the median values in each group. Figure 4**B** and 4**C** show the sex and the ECOG distribution in the test (green bars) and diffusion model-generated (salmon bars) data sets. Figure 4**D** and 4**E** show the univariate probability densities of weight and tumor volumes in the test (green bars) and diffusion model-generated (salmon bars) data sets. The regions of overlap are in the darker shade of brown. Figure 4F is a lower-triangular matrix of the difference correlogram, which reflects differences in the bivariate Spearman correlations between the generated and test data sets

The mean concentration profile of the diffusion-generated data closely matched the test data, with a relative error of 1.36% across the 13 time points (Fig. 4A). The smoothness ratio of adjacent concentration changes was 1.008 (diffusion/test), indicating preservation of PK plausibility without non-physiological discontinuities.

The univariate marginal distributions of the 13 sampled concentration timepoints showed extensive overlap between the test and diffusion-generated data (Supplementary Figure S4), with a mean KSD of 0.047 (Range: 0.041 for Cmax1 to 0.055 for Trough6). The binary covariates were also satisfactorily approximated, with absolute errors of 1.85% for sex and 1.40% for ECOG (Fig. 4B and C).

The bivariate Spearman correlation values between the variable pairs were preserved with a mean absolute difference of 0.012 and a maximum of 0.033 (Fig. 4F).

### TDM for generation of nivolumab PK trajectories

The TDM was trained for 2000 epochs and achieved a training loss of 0.010 in approximately 70 min on a single GPU.

**Fig. 5:**
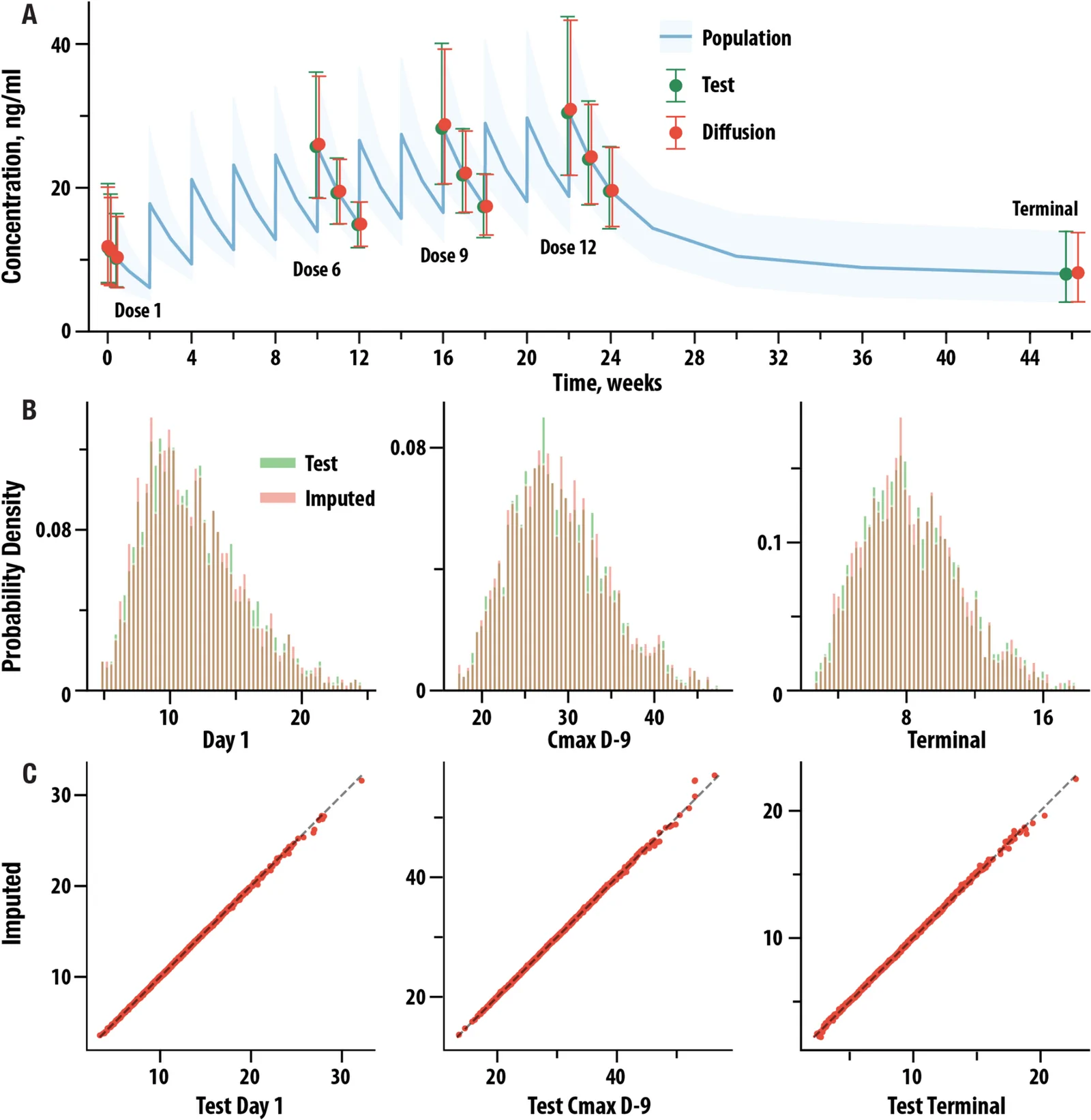
The lines in Figure 5**A** shows the median trajectory of nivolumab concentrations from the time-aware diffusion model for the test (green circles) and time-aware diffusion model (TDM) generated (salmon circles) data sets at the following time points: Cmax D-1 (Cycle 1 at end of infusion), Day 1, Day 3, Cmax D-6 (Cycle 6 at end of infusion), Mid Dose-6 (Mid D-6), Trough Dose-6 (Trough D-6), Cmax D-9 (Cycle 9 at end of infusion), Mid Dose-9 (Mid D-9), Trough Dose-9 (Trough D-9), Cmax D-12 (Cycle 12 at end of infusion), Mid Dose-12 (Mid D-12), Trough Dose-12 (Trough D-12), and Terminal phase concentrations. The test and generated medians were jittered to enable comparisons. The error bars around the sampled median points correspond to the 5^th^-95^th^ percentile confidence intervals for each group. The blue line and blue regions correspond to the median and 5^th^-95^th^ percentiles of nivolumab PK profiles from a full simulation. The histograms in Figure 5**B** show univariate probability density histograms for the Day 1, Cmax D-9, and Terminal phase concentration distributions from test data (green bars) and the imputed data from the time-aware diffusion model (salmon bars). Figure 5**C** is a visual predictive plot of imputed data from the time-aware diffusion model vs. test data for the Day 1, Cmax D-9, and Terminal phase concentration distributions. The dashed line is the identity line

The TDM-generated concentration profiles closely matched the test data across all 13 timepoints (Fig. 5A), with a mean trajectory relative error of 0.88% and a smoothness ratio of 0.992. The univariate marginal distributions showed a mean KSD of 0.028 across the 13 concentration timepoints (range: 0.017 for Cmax6 to 0.034 for Terminal). The binary covariates were exactly reproduced because the TDM was conditioned on real test covariates during generation. The bivariate correlation structure was preserved with a mean absolute correlation difference of 0.015.

In the imputation test, TDM accurately reconstructed masked concentrations from observed data (Fig. 5B and C). The mean absolute percentage error (MAPE) was 0.25% for Cmax D-9 (*r* > 0.999), 0.43% for Day 1 (*r* > 0.999), and 0.76% for the Terminal phase (*r* > 0.999). The KSD values of real and imputed distributions were ≤ 0.014 for all three time points. The imputation accuracy varied with the position of each timepoint in the global quantile-normalized space: Cmax D-9, which maps near the center of the pooled distribution, achieved the highest accuracy, while the Terminal phase, located in the lower tail, showed the largest imputation error. These results confirm that the TDM learned the temporal covariance structure of PK trajectories well enough to predict missing concentrations using neighboring observed time points and patient covariates.

### Comparison of exposure measures and covariate-exposure relationships

We assessed exposure measures and covariate-exposure relations for the TDM model. Trough concentrations have been widely used as an exposure metric in model-informed drug development (MIDD) plans for diverse drug products, in lieu of the area under the curve, which is difficult to measure in clinical settings [27]. We therefore focused on nivolumab trough concentrations as a relevant exposure metric for comparing DDPM results with established methods and examined their covariate relationships.

**Fig. 6:**
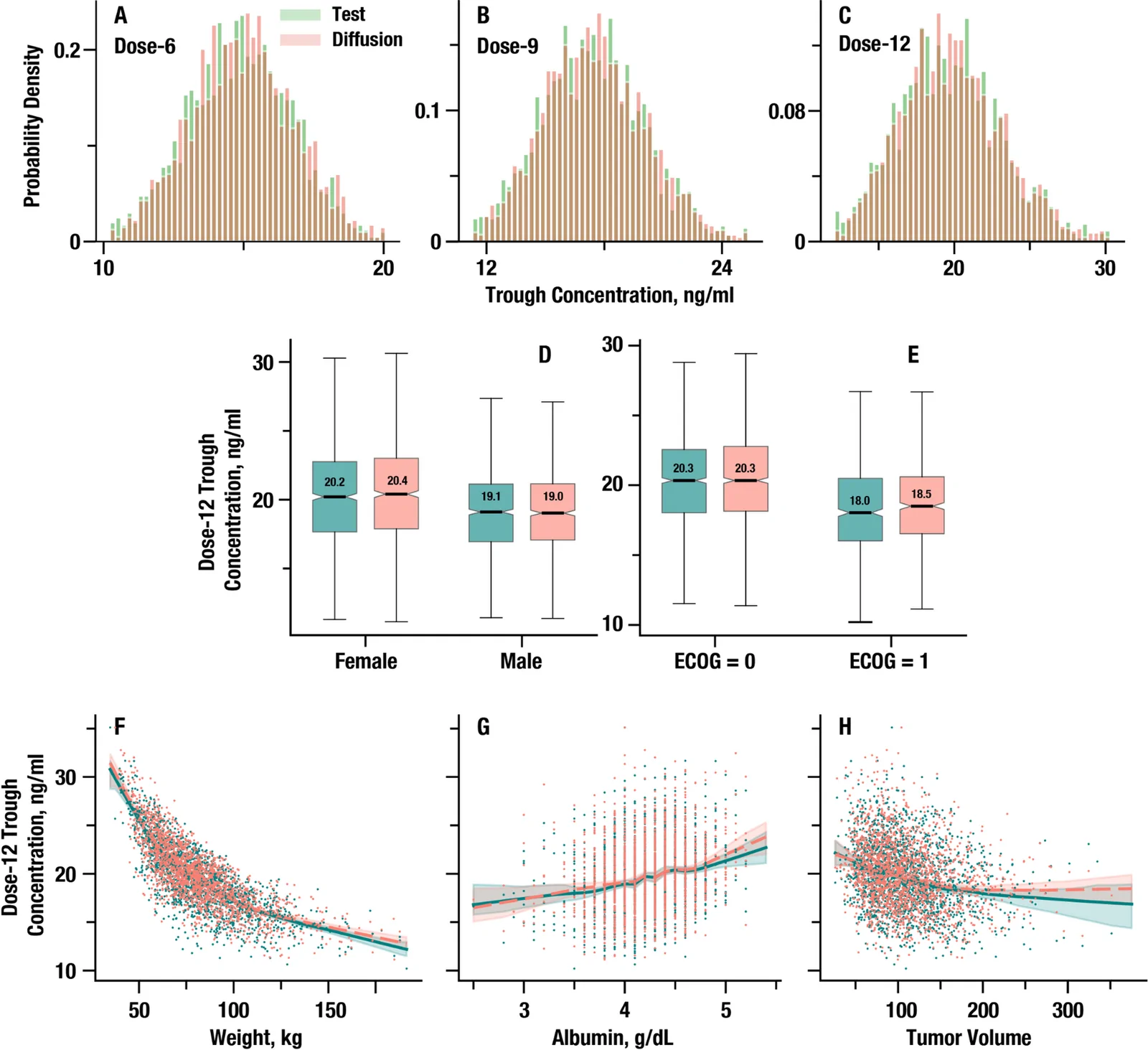
Figures 6**A**-**C** are probability density histograms of generated data for the trough concentrations (ng/ml) at Cycles 6, 9, and 12 from the time-aware diffusion model (teal bars) compared to test data (salmon bars). The darker regions of the histogram bars correspond to the regions of overlap. The boxplots show the dependence of Cycle 12 trough concentrations on sex (Figures 6**D**) and ECOG performance status (Figures 6**E**) for the test (teal) and generated (salmon) data sets. The central line in the box represents the median and the median values are labeled; the top and bottom edges of the box represent the 75th and 25th percentiles; the error bars represent the 1.5-fold interquartile range; and the filled circles represent outliers (beyond the 1.5-fold interquartile range). The notches are the comparison intervals around the median values. The scatter plots show the dependence of Cycle 12 trough concentrations on weight (Figure 6**F**), albumin levels (Figure 6**G**), and tumor volume (Figure 6**H**) for the test (teal points) and generated (salmon points) data sets. The lines (test: teal line; generated: dashed salmon line) represent the loess fit to the data; the shaded regions correspond to the 5^th^-95^th^ confidence intervals of the loess fit to the scatter plots

Figures 6A-C compare the probability density histograms of Cycles 6, 9, and 12 trough concentrations in the test and DDPM-generated datasets from the TDM analyses. The extensive overlap between the test and generated trough concentration histograms for Cycle 6 (KSD = 0.034), Cycle 9 (KSD = 0.020), and Cycle 12 (KSD = 0.034) indicates the potential utility of DDPM-based generative methods for exposure assessments. The box plots in Figs. 6D-E summarize the distribution of Cycle 12 trough concentrations by sex and ECOG performance status, and the loess plots in Figs. 6F-G show the dependence on WT, ALB, and TUM. The overlap between the box plots and loess lines is also consistent with a satisfactory approximation of the discrete- and continuous-covariate exposure relationships.

## Discussion

We evaluated a denoising diffusion probabilistic model for generative modeling of the joint distribution of a panel of 18 continuous biomarkers and 13 binary disease-status variables spanning renal, hepatic, diabetic, and cardiac conditions. The diffusion-generated data showed satisfactory agreement with the test data across univariate, bivariate, and multivariate evaluation criteria and demonstrated strength in reproducing the frequency distributions of categorical disease status variables.

The primary focus of our research was to assess the generative capabilities of diffusion models for virtual populations for clinical trial simulations, and we obtained a preliminary assessment of their utility for imputing missing time points with TDM. Our results suggest that diffusion models show promise for data generation and complement traditional pharmacometric methods, which are more useful for parameter estimation and compartmental simulations. Pharmacometricians might criticize diffusion models as “black boxes” because the encoder weights, which contain the learned computational representation of the high-dimensional joint distribution, cannot be interpreted. The structural parameters (e.g., micro rate constants) of compartmental pharmacometric models appear easier to interpret because they are explicitly specified by the user. However, the underlying PK time courses depend fundamentally on the system’s eigenvalues, which are hybrid macro rate constants involving multiple parameters interacting in more opaque ways. Pharmacometrics approaches are incapable of identifying dependencies not envisioned in the user’s model, leaving them susceptible to big blind zones in high-dimensional data settings.

We selected the ORQ normalization approach because it yields good approximations to the standard normal distribution across a diverse range of input distributions. Diffusion models rely on adding Gaussian noise during the forward process, and ORQ normalization ensures that the input data approximate this distributional assumption regardless of the original variable’s distribution. This preprocessing strategy differs from the logarithmic transform with min-max scaling in our earlier work with TVAE [6]. The ORQ transformation is invertible, and the original distribution can be recovered. An additional advantage of the ORQ method over min-max normalization is that the range of generated outputs is not bounded by the minimum and maximum values observed in the training set.

Several limitations of this study should be noted. We opted to implement listwise deletion of incomplete observations, which reduced the sample size from 23,825 to 17,480 subjects – because the missingness patterns were not random, and to avoid imputation artifacts. As listwise deletion reduces power and may bias the underlying joint distributions, we considered imputation as an alternative. We excluded mean value imputation because it reduces variance and ignores bivariate and higher-order dependencies; conditional mean value imputation was also excluded due to the challenges of identifying regression models with appropriate conditioning covariates. Multiple imputation with random forests, which considers nonlinear dependencies and multivariate interactions, and missingness-aware modeling could be attractive alternatives. The physiological biomarker and covariate evaluations were conducted using a single population-based data source (NHANES), but the approach could be strengthened by incorporating datasets from large disease registries and more complex datasets, including proteomic, metabolomic, and genetic data.

The PK data were generated using an established nivolumab population PK model, which may be criticized for being a structured and favorable simulation environment. The generalizability of the diffusion approach would be enhanced by additional real-world datasets that include challenging dependencies, sparsity, and measurement error, which would further strengthen confidence in the approach’s generalizability. The current evaluation assessed imputation at three predefined time points representing distinct PK phases. Future work could investigate more general missing-data scenarios, including random masking patterns, varying levels of missingness, and datasets with heterogeneous sampling schedules, and evaluate whether synthetic datasets preserve inferential properties relevant to pharmacometric workflows, such as population PK parameter recovery, model refitting accuracy, and covariate selection consistency. We also did not investigate neural ordinary differential equations (N-ODEs), which are compartmental models containing small neural networks, because they are primarily tools for time-course simulations rather than generation. Giacometti et al. [28]. demonstrated that N-ODEs incorporating covariates identified via Shapley additive explanations (SHAP) values outperformed NLME for long-term predictions of dalbavancin PK.

The diffusion model accurately reproduced disease status frequencies, with a mean absolute error of 0.31% points across 13 variables, including rare conditions such as dialysis and hepatitis. In contrast, TVAE showed a higher error rate (1.07% points) and systematically underestimated minority classes (e.g., kidney disease: 0.94% vs. 2.92% observed; liver disease: 0.14% vs. 1.95%). The diffusion model outperformed TVAE in 12 of 13 variables, likely due to its class-weighted loss and noise-prediction framework, which more effectively captures categorical distributions. However, we note that the diffusion model incorporated additional architectural optimizations, including residual connections, exponential moving average of weights, class-weighted loss, cosine annealing, and early stopping, that were not applied to the TVAE implementation. The possibility that the observed performance differences might be attributable to features of the diffusion framework’s implementation rather than to an intrinsic superiority of diffusion models over TVAE cannot be excluded.

While TVAE slightly outperformed diffusion on univariate distributions (KSD 0.054 vs. 0.065), it showed substantially worse performance in capturing variance (relative error 20.8% vs. 10.5%). For continuous variables, the diffusion model better preserved the correlation structure (mean absolute difference 0.033 vs. 0.091 for TVAE), suggesting improved modeling of joint dependencies. The conditional analyses demonstrated that the diffusion model captured disease–biomarker relationships, reproducing expected shifts (e.g., reduced GFR in kidney disease, elevated urinary albumin-to-creatinine ratio in diabetes). Greater variability was observed in small low-frequency subgroups (e.g., dialysis, *n* = 23 in the full dataset). Resampling methods, such as *k*-fold cross-validation, can be used to enhance the robustness of the generated data for such low-frequency variables. Low-frequency random variables exhibit high coefficients of variation, a limitation that affects all modeling approaches and is not unique to diffusion models. It should be noted that the frequency of rare events is often well described by a Poisson distribution.

Multivariate evaluation further supported diffusion model performance. A low MMD (0.006) indicated close agreement between the real and synthetic data distributions, and dimensionality reduction (t-SNE, UMAP, PCA) showed strong overlap with no evidence of mode collapse. In downstream tasks, synthetic data achieved 95.9% of the AUC obtained with real data, indicating preservation of clinically relevant signal.

We further evaluated diffusion models in the context of PK data, which differ from cross-sectional data due to temporal autocorrelation. The same DDPM architecture was applied to the simulated nivolumab PK dataset, which included concentration-time profiles at 13 sampled time points. Jiang et al. [29]. reported that a conditional generative adversarial network generating full PK profiles as single vectors outperformed time-series-aware models on PK datasets. The SDM achieved a mean KSD of 0.047 across concentration timepoints, preserved the Spearman correlation structure (mean absolute difference = 0.012), and closely approximated categorical proportions (sex error = 1.85%, ECOG error = 1.40%). The trajectory-level relative error of 1.36% and smoothness ratio of 0.998 indicate that the generated profiles are pharmacokinetically plausible.

The TDM, which incorporated temporal self-attention and global ordered-quantile normalization across the 13 sampling timepoints, provided complementary capabilities through its imputation mode. When three timepoints spanning distribution, steady-state, and elimination phases were masked, the model reconstructed Day 1 concentrations with an MAPE of 0.43% and Cmax D-9 concentrations with an MAPE of 0.25%. Terminal phase imputation was less accurate (MAPE = 0.76%, *r >* 0.999), reflecting the position of this timepoint in the tail of the global ordered quantile distribution, where the inverse transform amplifies small normalized-space errors. Analysis of the ordered-quantile mapping gradient confirmed that error amplification during inverse transformation was 2.6-fold greater at tail-region time points compared to centrally located time points.

Generative AI methods have raised widespread concerns about intellectual property and privacy, but they could also be useful for realizing the innovation potential of data sharing, meta-analytic methods, and open science. High-distributional-fidelity AI-generated datasets could help lower barriers to external scientific collaboration in the pharmaceutical industry, which relies on proprietary information and internal data silos to ensure market advantage. Nonetheless, oversight is critical because AI models may memorize rare patient characteristics or small subgroups, creating a re-identification risk.

In conclusion, the diffusion model demonstrated strong performance for generative modeling of both cross-sectional clinical biomarker panels and longitudinal pharmacokinetic concentration-time profiles. These results provide proof-of-concept evidence that diffusion models are a viable approach for synthesizing virtual patient populations that incorporate physiological biomarkers, disease states, and pharmacokinetic profiles relevant to drug dosing individualization.

## Supplementary Information

Below is the link to the electronic supplementary material.

Supplementary Material 1 (DOCX 3.47 MB)

## Data Availability

Public domain data were used for the research.

## Declarations

### Use of artificial intelligence

This is artificial intelligence (AI) research involving generative AI algorithms. AI-enabled tools such as Microsoft Word, Grammarly, and ChatGPT were used to enhance readability and language; tools such as Google and EndNote were employed to curate references, add citations, and generate the bibliography, and Illustrator was used to draw figures.
