# Supplementary material for: Diffusion models for virtual populations and pharmacometric simulations
Source: J Pharmacokinet Pharmacodyn. 2026 Jul 29;53(5):45. doi: 10.1007/s10928-026-10054-7 (PMC13421196; doi:10.1007/s10928-026-10054-7)
Supplement: Supplementary file 1 — Supplementary Material 1 (DOCX 3.47 MB) [file 10928_2026_10054_MOESM1_ESM.docx]

**SUPPLEMENTARY FILE**

**Diffusion Models for Virtual Populations and Pharmacometric Simulations**

Prathamesh Kishor Gadgil, Shamith Manjunath Poojari, and Murali Ramanathan

Artificial Intelligence & Clinical Pharmacology Laboratory, Department of Pharmaceutical Sciences, University at Buffalo, The State University of New York, Buffalo, NY, USA.

**CORRESPONDING AUTHOR:** Murali Ramanathan

355 Pharmacy, Department of Pharmaceutical Sciences

State University of New York, Buffalo, Buffalo, NY 14214-8033.

(716)-645-4846 and FAX 716-829-6569. E-mail Murali@Buffalo.Edu

**Running Head**: Diffusion models for pharmacometrics

**Keywords**: Diffusion models, physiological determinants of drug dosing, Generative AI, Artificial Intelligence, Pharmacometrics, Tabular VAE, Precision Medicine.

**SUPPLEMENTARY METHODS**

**Computed Biomarkers for Generative Diffusion Modeling of PK Covariates**

Several biomarkers in the PDODD panel were derived from primary biomarkers in NHANES using equations described in Titar and Ramanathan ([1](#_ENREF_1)).

Body surface area ($BSA$, m^2^) was calculated from $Weight (kg)$ and $Height (cm)$ using the Dubois and Dubois equation ([2](#_ENREF_2)):

$$BSA \left( m^{2} \right)=0.007184 {Weight (kg)}^{0.425}{Height (cm)}^{0.725}$$

Normalized waist circumference was ($WAISTMF$) was calculated from the waist circumference $(BMXWAIST ,cm)$ using divisors of 88 cm for women and 102 cm for men, which are the recommended treatment targets ([3](#_ENREF_3)):

$$WAISTMF=\frac{BMXWAIST\left( cm \right)}{88 \left( cm \right)} for women$$

$$WAISTMF=\frac{BMXWAIST\left( cm \right)}{102 \left( cm \right)} for men$$

Estimated plasma volume ($PLASMAVOL$, liters) was calculated from hematocrit ($LBXHCT$, %) and hemoglobin ($LBXHGB$, g/dl) with the Strauss formula ([4](#_ENREF_4)):

$$PLASMAVOL=\frac{(100-LBXHCT)}{LBXHGB}$$

Estimated glomerular filtration rate ($EGFR$, ml/(min1.73 m^2^)) was obtained from serum creatinine measurements using the CKI-EPI study 2021 formula ([5](#_ENREF_5)).

$$EGFR=142\times{0.9938}^{Age}\times min{({LBXSCR}/\kappa,1)}^{\alpha}\times max{({LBXSCR}/\kappa,1)}^{-1.2}\times\beta$$

In the equation: $LBXSCR$ is serum creatinine in mg/dl; $Age$ is in years; $\kappa$ is a constant that is 0.7 for females and 0.9 for males, $\alpha$ is a constant that is -0.241 for females and -0.302 for males; $\beta$ is a constant that is 1.012 for females and 1 for males ([5](#_ENREF_5)).

$RVALUE$ is a computed measure of liver function ([6](#_ENREF_6)) obtained from serum alanine aminotransferase ($LBXSATSI$) and serum alkaline phosphatase ($LBXSAPSI$) activity measurements in a standard complete metabolic panel (CMP).

$$RVALUE=\left( {LBXSATSI}/{{ULN}_{LBXSATSI}} \right)/\left( {LBXSAPSI}/{{ULN}_{LBXSAPSI}} \right)$$

${ULN}_{LBXSATSI}$ and ${ULN}_{LBXSAPSI}$, the upper limits of normal (ULN) for alanine aminotransferase and alkaline phosphatase, respectively. ${ULN}_{LBXSATSI}$ was set to 29 IU/L for males and 22 IU/L for females ([7](#_ENREF_7)) whereas ${ULN}_{LBXSAPSI}$ for the different racial groups was based on Gonzalez *et al*. ([8](#_ENREF_8)) as described in ([9](#_ENREF_9)).

The risk of drug-induced liver injury (DILI) is an important safety consideration in drug development and utilization. We used $DILIVALUE$, a measure of the DILI risk that was based on the work of Diaz-Robles *et al*. ([10](#_ENREF_10)) who identified an algorithm based on aspartate aminotransferase ($LBXSASSI$), bilirubin ($LBXSTB$), and the ratio of aspartate aminotransferase to alanine aminotransferase that improved on Hy’s law ([11](#_ENREF_11)):

$$DILIVALUE=\frac{1}{17.3}\left( \frac{LBXSASSI}{{ULN}_{LBXSASSI}} \right)\times\frac{1}{6.6}\left( \frac{LBXSTB}{{ULN}_{LBXSTB}} \right)\times\frac{1}{1.5}\left( \frac{LBXSASSI}{LBXSATSI} \right)$$

${ULN}_{LBXSASSI}$ and ${ULN}_{LBXSTB}$, are the upper limits of normal for aspartate aminotransferase, and bilirubin, respectively. Based on Sohn *et al*. ([12](#_ENREF_12)), ${ULN}_{LBXSASSI}$was set to 32 IU/L for men and 26 IU/L for women; ${ULN}_{LBXSTB}$ was set to 2 mg/dl based on Perlstein *et al*. ([13](#_ENREF_13)). There were a few zeroes in $LBXSTB$, the zero values were set to a small positive number (0.01), which was 2-fold lower than the next lowest measured value.

Hepatic steatosis index (HSI) was calculated as follows ([14](#_ENREF_14)):

$$HSI=2\times\left( \frac{LBXSATSI}{LBXSASSI} \right)+BMI+2\left( \text{if diabetic} \right)+2(\text{if female})$$

Average urine flow rate ($URDFLOW$, ml/min) was calculated using NHANES guidelines ([15](#_ENREF_15)).

The systemic immune-inflammation index (SII) was calculated using ([16](#_ENREF_16)):

$$SII=\frac{Platelet Count\times Neutrophil Count}{Lymphocyte Count}$$

Active hepatitis B virus (HBV) infection status was a binary variable that was set to unity for anti-HBV core antigen antibody (anti-HBc Ab, *LBXHBV*) positive subjects who tested positive for HBV surface antigen (HBsAg, LBDHBG) and 2 for anti-HBc Ab tested subjects not meeting the criterion. Active hepatitis C virus (HCV) infection status was a binary variable that was set to unity for anti-HCV screening antibody (anti-HCV Ab) positive subjects who tested positive for HCV-RNA (*LBXHCR*) and 2 for anti-HCV Ab screening antibody subjects not meeting the criterion.

**Renal Status:** Renal disease status was coded as two binary variables *Kidney Disease* and *Dialysis*. The *Kidney Disease* status variable was computed from the responses to NHANES variables KIQ022 (Ever told you had weak/failing kidneys?), and the *Dialysis* status variable from KIQ025 (Received dialysis in the past 12 months?).

**Hepatic Status:** Liver disease status was coded as four binary variables: *Active Liver Disease*, *Past Liver Disease*, *Active Hepatitis B*, and *Active Hepatitis C*. The *Past Liver Disease* status variable was computed from the responses to NHANES variables MCQ160L (Ever told you had any liver condition?); the *Active Liver Disease* was obtained from MCQ170L (Do you still have a liver condition?). *Active Hepatitis B* and *Active Hepatitis C* variables were computed as previously described ([9](#_ENREF_9)). Individuals were categorized as having an active hepatitis B infection positive status (HBV) if they were positive for anti-HBV core antigen antibody (anti-HBc Ab, LBXHBV), and positive for HBV surface antigen (HBsAg, LBDHBG). Individuals were categorized as having active hepatitis C (HCV) infection positive status if they were positive for anti-HCV screening antibody (anti-HCV Ab) and positive for HCV-RNA (LBXHCR).

**Diabetes Status:** Diabetes disease status was coded with three binary variables: *Diabetes*, *Prediabetes*, and *Insulin Use*. The *Diabetes* variable was computed from the responses to NHANES variables DIQ010 (Doctor told you have diabetes?); Insulin Use from DIQ050 (Taking insulin now?), and *Prediabetes* from DIQ160 (Ever told you have prediabetes?). Borderline diabetes was categorized as pre-diabetes.

**Cardiac Status:** Cardiac disease status variable was coded with four binary variables: *Congestive Heart Fail*ure (CHF), *Coronary Heart Disease* (CHD), *Angina Pectoris*, and *Heart Attack* from the responses to NHANES variables MCQ160B (Ever told you had congestive heart failure?), MCQ160C (Ever told you had coronary heart disease?), MCQ160D (Ever told you had angina pectoris?), and MCQ160E (Ever told you had heart attack?).

**Nivolumab Population Pharmacokinetic Model for Generative Diffusion Modeling of PK Trajectories**

**Structural Model:** The reference population pharmacokinetic (PopPK) model for nivolumab was adapted from the published two-compartment models described by Bajaj *et al*. ([17](#_ENREF_17)) and Liu *et al*. ([18](#_ENREF_18)), and implemented in R using the *nlmixr2* package ([19](#_ENREF_19)) Nivolumab pharmacokinetics were characterized by a two-compartment linear disposition model with zero-order intravenous infusion input and first-order elimination, parameterized in terms of systemic clearance ($CL$), central volume of distribution ($V1$), peripheral volume of distribution ($V2$), and intercompartmental clearance ($Q$).

The system of ordinary differential equations governing drug disposition is:

$$\frac{d}{dt}\left( \text{central} \right)=- k_{el}\cdot\text{central}-k_{12}\cdot\text{central}+k_{21}\cdot\text{peripheral}$$

$$\frac{d}{dt}(\text{peripheral})=k_{12}\cdot\text{central}-k_{21}\cdot\text{peripheral}$$

with predicted plasma concentration defined as:

$$C_{p}=\frac{\text{central}}{V_{1}}$$

and first-order rate constants:

$$k_{el}=\frac{CL(t)}{V_{1}},k_{12}=\frac{Q}{V_{1}},k_{21}=\frac{Q}{V_{2}}$$

**Time-Varying Clearance:** A defining PK feature of nivolumab is a systematic change in clearance over the treatment course, linked to post-treatment disease dynamics including tumor burden reduction and reversal of cancer-associated cachexia ([17](#_ENREF_17), [18](#_ENREF_18)). This was incorporated using a sigmoid Emax (Hill) function applied multiplicatively to the individual baseline clearance, consistent with the time-dependent PK (TDPK) model structure described by Liu et al. ([18](#_ENREF_18)):

$$CL(t)=CL_{0}\cdot\exp\text{ }\left( \frac{CL_{max}\cdot t^{\gamma}}{CL_{50}^{\gamma}+t^{\gamma}} \right)$$

where ${CL}_{0}$ is the individual baseline clearance incorporating all covariate effects and between-subject variability (BSV); ${CL}_{max}$ is the maximum fractional change in clearance on the log scale; ${CL}_{50}$ is the time at which 50% of the maximum clearance change has occurred; and $\gamma$ is the Hill coefficient governing the sigmoidicity of the time-effect relationship. Under this parameterization, clearance increases monotonically from its baseline value toward an asymptote of ${CL}_{0}e^{{CL}_{max}}$ as $t\to\infty.$

The parameters ${CL}_{max}$, ${CL}_{50}$, and $\gamma$ were fixed to their published values from Bajaj *et al*. (2017) - 0.22, 30 days, and 3.15.

**Covariate Model:** Five patient-level covariates were incorporated into the structural model based on the nivolumab PK model in Bajaj *et al*. and Liu et al. ([17](#_ENREF_17), [18](#_ENREF_18)). The individual baseline clearance, inclusive of all covariate effects and BSV, was parameterized as:

$$CL_{0}=\theta_{CL}\cdot\left( \frac{WT}{80} \right)^{0.75}\cdot\left( \frac{ALB}{4} \right)^{\theta_{ALB}}\cdot e^{\theta_{ECOG}\cdot ECOG}\cdot\left( \frac{TUM}{100} \right)^{\theta_{TUM}}\cdot e^{\eta_{CL}}$$

The allometric scaling exponent for body weight on clearance (0.75) was fixed.

Serum albumin was included as a continuous covariate on clearance based on the sensitivity analysis by Bajaj *et al*. ([17](#_ENREF_17)), which demonstrated a clinically meaningful effect exceeding 20% despite its exclusion from the final reduced model due to missing data across trials.

Baseline tumor burden was incorporated following Liu *et al*. ([18](#_ENREF_18)), who demonstrated that longitudinal tumor size significantly covaries with time-varying clearance, with tumor dynamics partially explaining the reduced clearance during treatment. ECOG performance status was included as a binary covariate (0 or 1) consistent with both published models ([17](#_ENREF_17), [18](#_ENREF_18)).

Central and peripheral volumes were parameterized as:

$$V_{1}=\theta_{V_{1}}\cdot\left( \frac{WT}{80} \right)^{1.0}\cdot e^{\theta_{SEX}\cdot SEX}\cdot e^{\eta_{V_{1}}}$$

$$V_{2}=\theta_{V_{2}}\cdot\left( \frac{WT}{80} \right)^{1.0}$$

The allometric exponent for volume was fixed at 1, consistent with standard scaling for monoclonal antibodies. Intercompartmental clearance $Q$ was modeled as a fixed population parameter with no covariate relationships. Reference covariate values were $WT$ = 80 kg, $ALB$ = 4 g/dL, and $TUM$ = 100 mm. $SEX$ was coded as 0 (Female) or 1 (Male).

**Model Parameters:** The population typical values used for simulation are shown in Supplementary Table S1. Parameters designated as fixed were held constant.

**Residual Error Model:** A proportional residual error model was used:

$$C_{obs}=C_{p}\cdot(1+\varepsilon),\varepsilon\sim\mathcal{N}(0,\text{ }\sigma_{prop}^{2})$$

with an initial proportional error estimate of 10% CV ($\sigma_{prop}$ = 0.10). BSV on CL and V1 was modeled using a log-normal distribution with initial variance estimates of 0.04, corresponding to approximately 20% CV.

**Covariate Population:** Virtual patient covariates for body weight ($WT$) and serum albumin ($ALB$) were sampled from the National Health and Nutrition Examination Survey (NHANES) database, providing a representative distribution of these variables from a real-world adult US population. Body weight was derived from the NHANES anthropometric variable BMXWT and serum albumin from the laboratory variable LBXSAL. Sex was encoded as a binary variable $SEX$ (1 = male, 0 = female) derived from the NHANES gender field RIAGENDR.

A random sample of 12,000 subjects was drawn with replacement to constitute the simulation population. This sample size was selected to provide sufficient training data for the generative model.

ECOG performance status and baseline tumor burden were not available in NHANES and were generated synthetically at the subject level to replicate distributions reported in the nivolumab clinical trial population included in Bajaj et al. (2017). ECOG was drawn from a Bernoulli distribution with a $P(ECOG=1)$ probability of 0.35, reflecting the proportion of patients with impaired performance status in that dataset. Tumor burden was sampled from a log-normal distribution with meanlog = log(100) mm and sdlog = 0.4, corresponding to a median baseline tumor burden of 100 mm with realistic right-skewed variability, broadly consistent with baseline tumor size distributions reported across the nivolumab phase III trials.

**Dosing Regimen:** Nivolumab was administered as a weight-based intravenous infusion at 3 mg/kg every two weeks (Q2W) for 12 consecutive doses, consistent with the clinical dosing regimen used across the phase I–III trials included in the Bajaj *et al*. (2017). PopPK analysis. Each dose was delivered as a 1-hour infusion. Individual dose amounts were computed as $3{WT}_{i}$ mg per patient, where ${WT}_{i}$ is the individual body weight drawn from the NHANES sample.

**Sampling Schedule:** A 13-timepoint sparse sampling schedule was defined to capture clinically relevant pharmacokinetic features across the full treatment course and into the terminal elimination phase. Timepoints were selected to represent the Cycle 1 peak and early distribution phase, steady-state trough, mid-interval, and peak concentrations at Cycles 6, 9, and 12, and a terminal elimination sample at Day 322, approximately 154 days after the final dose. The sampling time points are listed in Table S2.

Simulations were executed using *nlmixr2*.

**SUPPLEMENTARY TABLES**

**Table S1.** Population PK model parameters.

| **Parameter** | **Description** | **Value** | **Units** | **Status** |
| --- | --- | --- | --- | --- |
| $\theta_{CL}$ | Baseline clearance | 0.200 | L/day | Estimated |
| $\theta_{V1}$ | Central volume | 20.0 | L | Estimated |
| $\theta_{V2}$ | Peripheral volume | 150.0 | L | Estimated |
| $\theta_{Q}$ | Intercompartmental clearance | 0.750 | L/day | Estimated |
| ${CL}_{max}$ | Maximum fractional CL change | 0.220 | – | Fixed |
| ${CL}_{50}$ | Time to 50% CL change | 30.0 | days | Fixed |
| $\gamma$ | Hill coefficient | 3.15 | – | Fixed |
| ${CL}_{WT}$ | WT allometric exponent on CL | 0.750 | – | Fixed |
| $V_{WT}$ | WT allometric exponent on V | 1.000 | – | Fixed |
| $\theta_{ALB}$ | Albumin effect on CL | -0.600 | – | Estimated |
| $\theta_{ECOG}$ | ECOG effect on CL | 0.250 | – | Estimated |
| $\theta_{TUM}$ | Tumor burden effect on CL | 0.300 | – | Estimated |
| $\theta_{SEX}$ | Sex effect on V1 | 0.100 | – | Estimated |
| $\omega_{CL}^{2}$ | BSV variance on CL | 0.040 | – | Estimated |
| $\omega_{V1}^{2}$ | BSV variance on V1 | 0.040 | – | Estimated |
| $\sigma_{prop}$ | Proportional residual error | 0.100 | – | Estimated |

**Table S2.** Pharmacokinetic sampling schedule.

| **Label** | **Time (days)** | **PK feature** |
| --- | --- | --- |
| Cmax D-1 | 0.0417 (1 hour) | Cycle 1 at end of infusion |
| Day 1 | 1.0 | Cycle 1, day 1 |
| Day 3 | 3.0 | Cycle 1, day 3 |
| Cmax D-6 | 70.0417 | Cycle 6 at end of infusion |
| Mid D-6 | 77.0 | Cycle 6 mid-interval |
| Trough D-6 | 84.0 | Cycle 6 trough |
| Cmax D-9 | 112.0417 | Cycle 9 at end of infusion |
| Mid D-9 | 119.0 | Cycle 9 mid-interval |
| Trough D-9 | 126.0 | Cycle 9 trough |
| Cmax D-12 | 154.0417 | Cycle 12 at end of infusion |
| Mid D-12 | 161.0 | Cycle 12 mid-interval |
| Trough D-12 | 168.0 | Cycle 12 trough/end of treatment |
| Terminal | 322.0 | Terminal elimination phase |

**SUPPLEMENTARY FIGURE LEGENDS**

**Figure S1.** Pairs panel plot of the ordered quantile normalized logarithm-transformed values of eight continuous variables: weight (BMXWT, body surface area (BSA), estimated glomerular filtration rate (EGFR), platelet count (LBXPLTSI), red blood cell count (LBXRBCSI), albumin (LBXSAL), and urine albumin-to-creatinine ratio (URDACT), and urine flow. All variables were log transformed and ordered quantile normalized logarithm-transformed values. The diagonal contains the univariate probability density functions. The lower triangular region represents the bivariate scatter plot of the variables along the row and column as a contour plot (green circles: test data, red circles: diffusion model-generated data). The upper triangular region shows correlation coefficients for the test (green font) and diffusion model-generated data (red font) of the variables along the row and column.

**Figure S2.** The box plots show the dependence of ordered quantile normalized logarithm-transformed values of estimated glomerular filtration rate (EGFR, Figures A-H), urine albumin-creatinine ratio (Urine ACR, Figures I-P), and plasma volume (Figure Q-X) in the test data (green boxes) and the diffusion model-generated data (red boxes) for the groups on dialysis, kidney disease, diabetes, insulin use, active liver disease, past liver disease, congestive heart failure (CHF), and coronary heart disease (CHD) status. The central line in the box represents the median; the top and bottom edges of the box represent the 75th and 25th percentiles; the error bars represent the 1.5-fold inter-quartile range; and the filled circles represent outliers (beyond the 1.5-fold inter-quartile range). The notches are the comparison intervals around the median values. Abbreviations: CHF: Congestive heart failure; CHD: Coronary heart disease.

**Figure S3.** Univariate probability density histograms of the ordered quantile normalized values of continuous variables for the test data and five generated datasets. Abbreviations: RIDAGEYR: Age; LBXWT: Body weight; WAISTMF: Normalized waist circumference; PLASMAVOL: Plasma volume; SII: Systemic inflammation index; R-VALUE: Hepatic R-value; HSI: Hepatic steatosis index; URDFLOW: Urine flow rate; URXUCR: Urine creatinine; URDACT: Urine albumin-creatinine ratio; LBXRBCSI: Red blood cell count; LBXPLTSI: Platelet count; LBDLYMNO: Lymphocyte count; LBDNENO: Neutrophil count; BSA: Body surface area; LBXSAL: Serum albumin; DILI: Drug-induced liver injury; EGFR: Estimated glomerular filtration rate.

**Figure S4.** Univariate probability density of nivolumab concentrations in the test (green bars) and diffusion model-generated (salmon bars) data sets for the sequence-dependent model. The regions of overlap are in the darker shade of brown. The following time points were sampled: Cmax D-1, Day 1, Day 3, Cmax D-6, Mid Dose-6, Trough Dose-6, Cmax D-9, Mid Dose-9, Trough Dose-9, Cmax D-12, Mid Dose-12, Trough Dose-12, and Terminal phase concentrations.

**FIGURE S1**

**
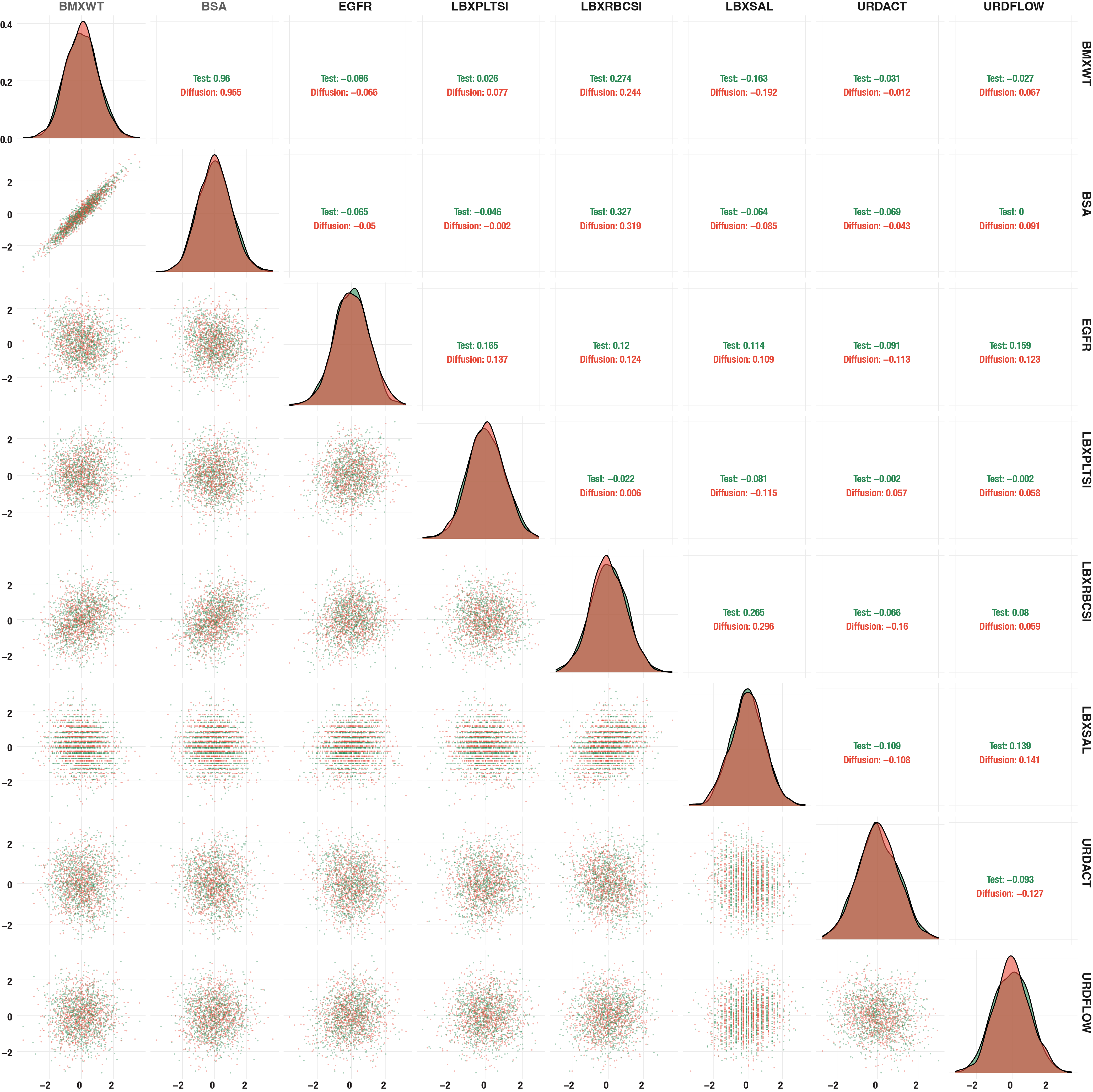
**

**FIGURE S2**


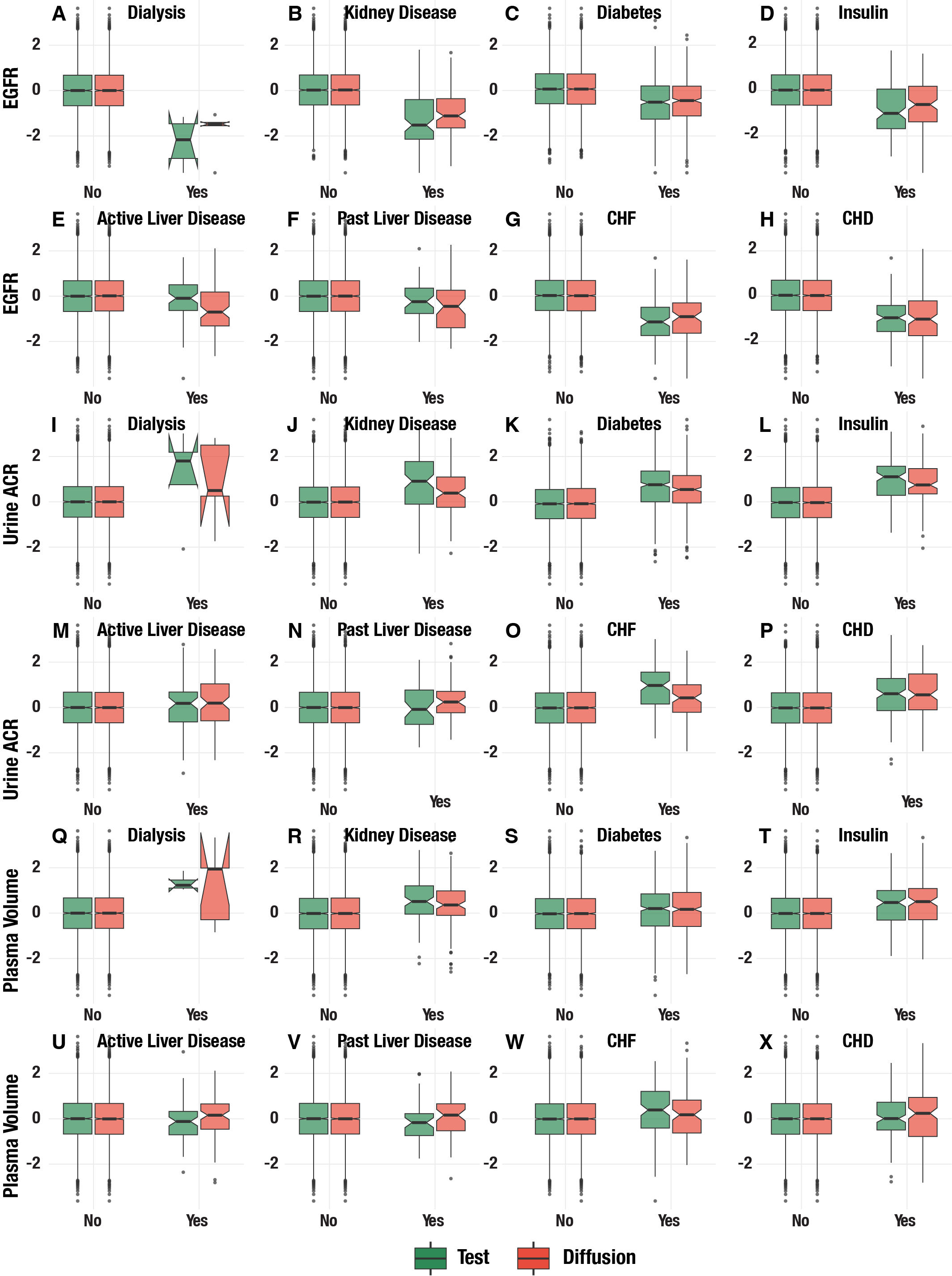


**FIGURE S3**


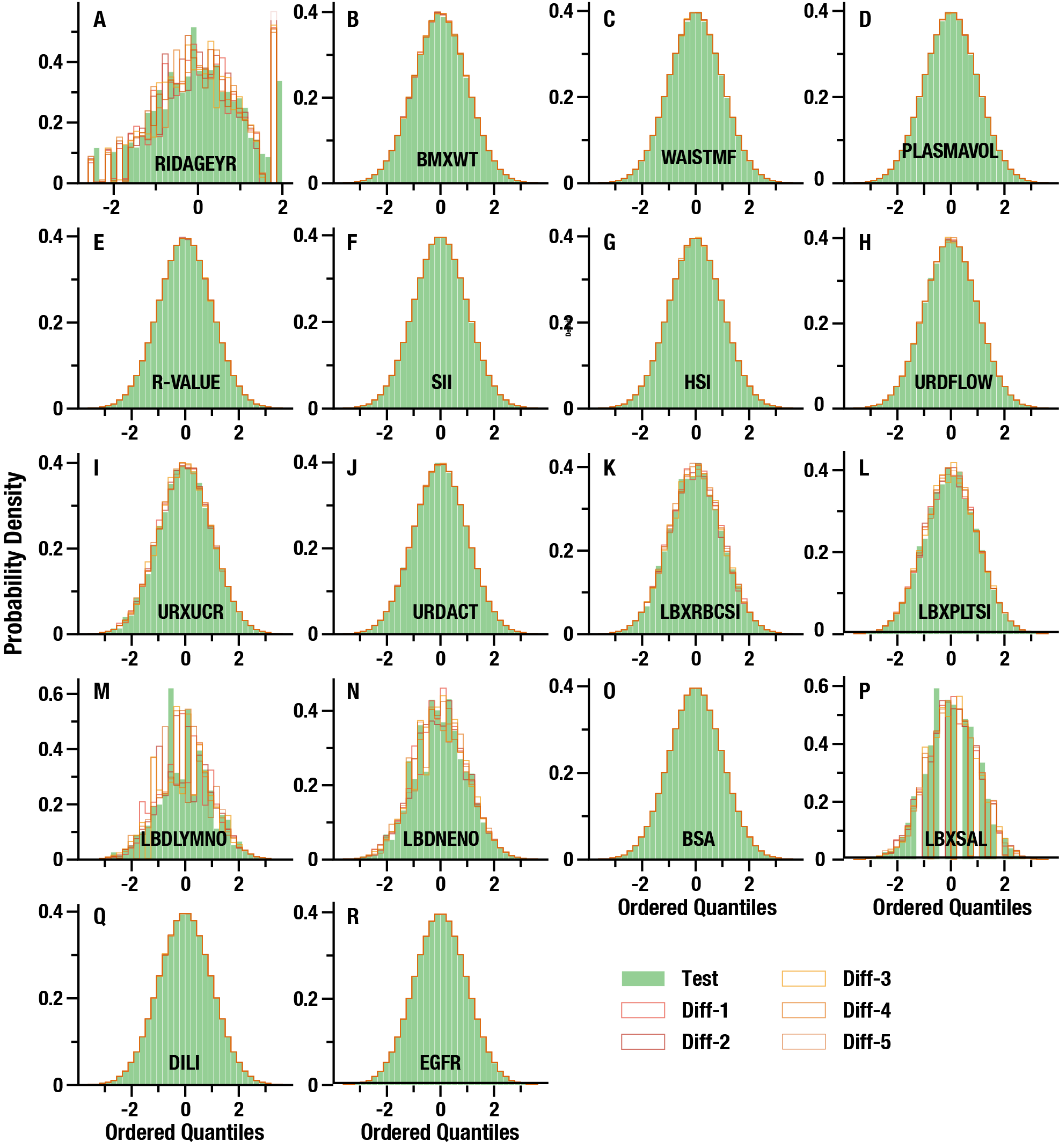


**FIGURE S4**


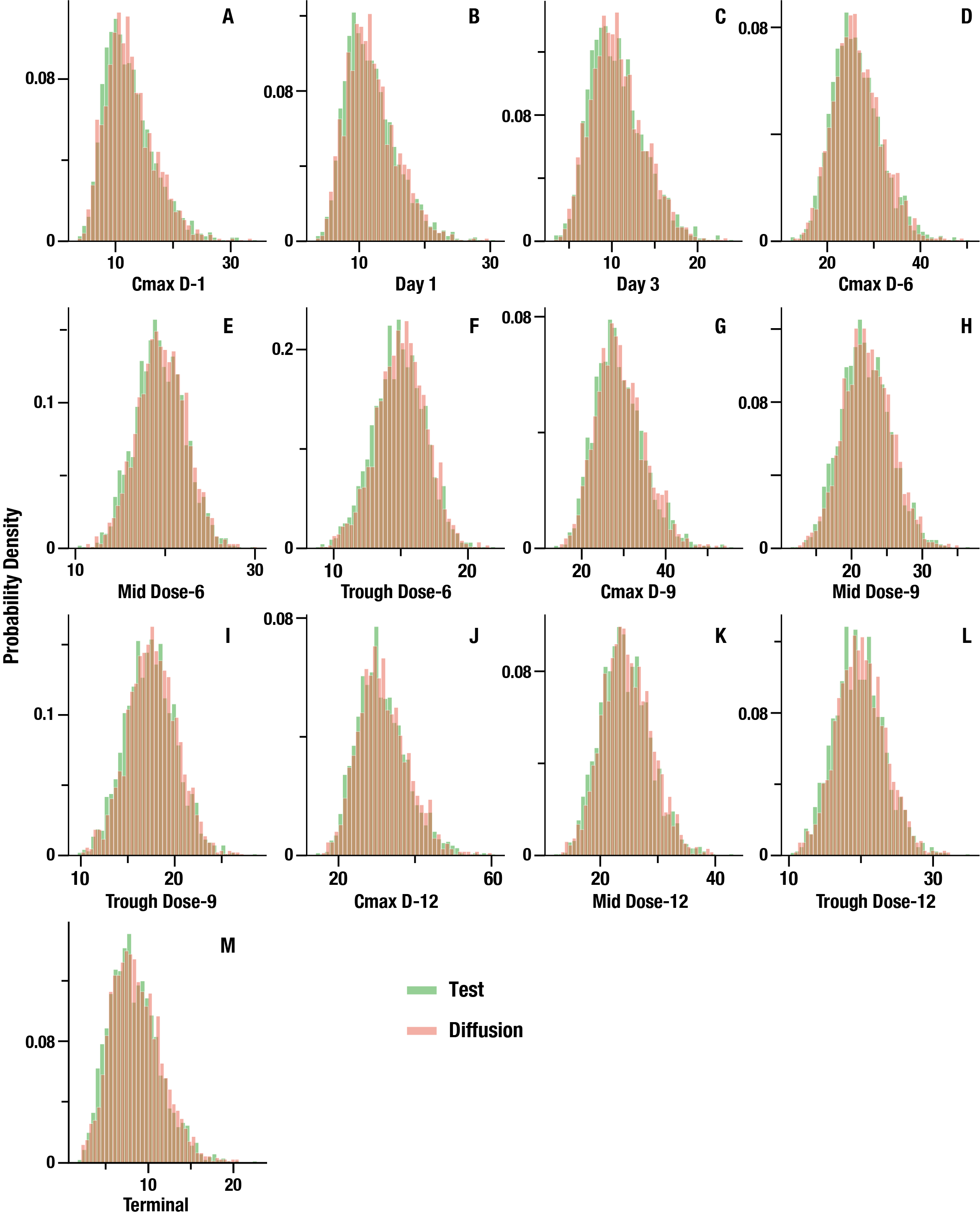


**REFERENCES**

(1) Titar, R.R. & Ramanathan, M. Variational autoencoders for generative modeling of drug dosing determinants in renal, hepatic, metabolic, and cardiac disease states. *Clin Transl Sci* **17**, e13872 (2024).

(2) Dubois, D. & Dubois, E.F. A formula to estimate the approximate surface area if height and weight be known. *Arch Intern Med* **17**, 863-71. (1916).

(3) Ross, R. *et al.* Waist circumference as a vital sign in clinical practice: a Consensus Statement from the IAS and ICCR Working Group on Visceral Obesity. *Nat Rev Endocrinol* **16**, 177-89 (2020).

(4) Strauss, M.B., Davis, R.K., Rosenbaum, J.D. & Rossmeisl, E.C. Water diuresis produced during recumbency by the intravenous infusion of isotonic saline solution. *J Clin Invest* **30**, 862-8 (1951).

(5) Inker, L.A. *et al.* New Creatinine- and Cystatin C-Based Equations to Estimate GFR without Race. *N Engl J Med* **385**, 1737-49 (2021).

(6) Chalasani, N.P. *et al.* ACG Clinical Guideline: the diagnosis and management of idiosyncratic drug-induced liver injury. *Am J Gastroenterol* **109**, 950-66; quiz 67 (2014).

(7) Ruhl, C.E. & Everhart, J.E. Upper limits of normal for alanine aminotransferase activity in the United States population. *Hepatology* **55**, 447-54 (2012).

(8) Gonzalez, H. *et al.* Normal alkaline phosphatase levels are dependent on race/ethnicity: NationalGEP Health and Nutrition Examination Survey data. *BMJ Open Gastroenterol* **7**, (2020).

(9) Nair, R., Mohan, D.D., Setlur, S., Govindaraju, V. & Ramanathan, M. Generative models for age, race/ethnicity, and disease state dependence of physiological determinants of drug dosing. *J Pharmacokinet Pharmacodyn* **50**, 111-22 (2023).

(10) Robles-Diaz, M. *et al.* Use of Hy's law and a new composite algorithm to predict acute liver failure in patients with drug-induced liver injury. *Gastroenterology* **147**, 109-18 e5 (2014).

(11) Zimmerman, H.J. Drug-induced liver disease. *Drugs* **16**, 25-45 (1978).

(12) Sohn, W. *et al.* Upper limit of normal serum alanine and aspartate aminotransferase levels in Korea. *J Gastroenterol Hepatol* **28**, 522-9 (2013).

(13) Perlstein, T.S., Pande, R.L., Creager, M.A., Weuve, J. & Beckman, J.A. Serum total bilirubin level, prevalent stroke, and stroke outcomes: NHANES 1999-2004. *Am J Med* **121**, 781-8 e1 (2008).

(14) Lee, J.H. *et al.* Hepatic steatosis index: a simple screening tool reflecting nonalcoholic fatty liver disease. *Dig Liver Dis* **42**, 503-8 (2010).

(15) National Health and Nutrition Examination Survey. National Health and Nutrition Examination Survey: NHANES 2015-2016 Overview. (ed. National Center for Health Statistics) (Centers for Disease Control, 2015).

(16) Song, Y., Guo, W., Li, Z., Guo, D., Li, Z. & Li, Y. Systemic immune-inflammation index is associated with hepatic steatosis: Evidence from NHANES 2015-2018. *Front Immunol* **13**, 1058779 (2022).

(17) Bajaj, G., Wang, X., Agrawal, S., Gupta, M., Roy, A. & Feng, Y. Model-Based Population Pharmacokinetic Analysis of Nivolumab in Patients With Solid Tumors. *CPT Pharmacometrics Syst Pharmacol* **6**, 58-66 (2017).

(18) Liu, C. *et al.* Association of time-varying clearance of nivolumab with disease dynamics and its implications on exposure response analysis. *Clin Pharmacol Ther* **101**, 657-66 (2017).

(19) Hooijmaijers, R., Fidler, M., Denney, B. & Vedantham, K. Package ‘nlmixr2lib’: A model library for ‘nlmixr2’. (2026).
